# Comparing the clinical utility of single-shot, readout-segmented and zoomit echo-planar imaging in diffusion-weighted imaging of the kidney at 3 T

**DOI:** 10.1038/s41598-022-16670-w

**Published:** 2022-07-20

**Authors:** Wenguang Liu, Hui Liu, Simin xie, Ismail Bilal Masokano, Yu Bai, Xiao Wang, Linhui Zhong, Yi Wu, Jilin Nie, Gaofeng Zhou, Yigang Pei, Wenzheng Li

**Affiliations:** 1 Department of Radiology, Xiangya Hospital, Central South University, No. 87 Xiangya Rd., Kai Fu District, Changsha, 410008 Hunan People’s Republic of China; 2 National Clinical Research Center for Geriatric Disorders, Xiangya Hospital, Central South University, Changsha, 410008 Hunan People’s Republic of China; 3 Department of Radiology, The Third Xiangya Hospital, Central South University, Changsha, 410013 Hunan People’s Republic of China

**Keywords:** Kidney, Magnetic resonance imaging

## Abstract

We compared the clinical utility of single-shot echo-planar imaging (SS-EPI) using different breathing schemes, readout-segmented EPI and zoomit EPI in the repeatability of apparent diffusion coefficient (ADC) measurements, cortico-medullary contrast to noise ratio (c-mCNR) and image quality. In this institutional review board-approved prospective study, some common clinically applicable diffusion-weighted imaging (b = 50, 400, 800 s/mm^2^) of kidney on 3.0 T MRI were performed on 22 volunteers using SS-EPI with breath-hold diffusion-weighted imaging (BH-DWI), free-breathing (FB-DWI), navigator-triggered (NT-DWI) and respiratory-triggered (RT-DWI), readout-segmented DWI (RS-DWI), and Zoomit DWI (Z-DWI). ADC and c-mCNR were measured in 12 anatomic locations (the upper, middle, and lower pole of the renal cortex and medulla), and image quality was assessed on these DWI sequences. A DWI with the optimal clinical utility was decided by systematically assessing the ADC repeatability, c-mCNR and image quality among the DWIs. For ADC measurements, Z-DWI had an excellent intra-observer agreement (intra-class correlation coefficients (ICCs): 0.876–0.944) and good inter-observer agreement (inter-class ICCs: 0.798–0.856) in six DWI sequences. Z-DWI had the highest ADC repeatability in most of the 12 anatomic locations of the kidneys (mean ADC absolute difference: 0.070–0.111 × 10^−3^ mm^2^/s, limit of agreement: 0.031–0.056 × 10^−3^ mm^2^/s). In all DWIs, Z-DWI yielded a slightly higher c-mCNR than other DWIs in most representative locations (*P* > 0.05), which was significantly higher than BH-DWI and FB-DWI in the middle pole of both kidneys and the upper pole of the left kidney (*P* < 0.05). In addition, Z-DWI yielded image quality that was similar to RT-DWI and NT-DWI (*P* > 0.05) and superior to BH-DWI, FB-DWI and RS-DWI (*P* < 0.05). Our results suggest that Z-DWI provides the highest ADC reproducibility, better c-mCNR and good image quality on 3.0 T MRI, making it the recommended sequence for clinical DWI of the kidney.

## Introduction

Diffusion-weighted imaging (DWI) is a sensitive tool and an attractive technique to assess the so-called Brownian motion, which obtains not only anatomic and structural information but also qualitative and quantitative data of the kidney. Furthermore, it is a non-invasive examination and easy to perform on patients without using a contrast medium, which is essential for patients with renal dysfunction to avoid nephrogenic systemic fibrosis^1,2^.

At present, some DWI sequences have widely been used to diagnose renal diseases and evaluate the renal function in a large number of acute and/or chronic kidney diseases and various renal tumors^3–7^, including breath-hold DWI (BH-DWI)^8^, free-breathing DWI (FB-DWI)^9^, navigator-triggered DWI (NT-DWI)^10^, respiratory-triggered DWI (RT-DWI)^11^, readout-segmented DWI (RS-DWI)^12^, and zoomit-DWI (Z-DWI)^13^. Zoomit-DWI is a reduced field-of-view (rFOV) single-shot DWI sequence, which is also called as field-of-view optimized and constrained undistorted single-shot DWI (FOCUS DWI) in GE and zonal oblique multislice (ZOOM) in Philips^14,15^. Compared with conventional single-shot DWI (BH-DWI, FB-DWI, NT-DWI and RT-DWI), Z-DWI can reduce the interference of gastrointestinal motility and gas artifact on the kidney through small field of view local excitation^13^, and RS-DWI can reduce deformation artifact through staggered acquisition in frequency or phase encoding direction^12^. In the process, the apparent diffusion coefficient (ADC), which represents the mobility of water molecules within the tissue, is a vital quantitative imaging parameter and a diagnostic & therapeutic biomarker for patients with renal diseases. However, many factors, including gastrointestinal peristalsis, breathing and cardiac pulsations, can affect the accurate measurements of ADCs, cortico-medullary contrast to noise ratio(c-mCNR) and even the image quality^16–18^.

Thus, reliable measurement of ADCs, sufficient c-mCNR and good image quality are essential in assessing renal function and monitoring therapeutic effects using the DWI sequence. Currently, most studies on ADC reliability were focused on the liver, with only a few studies focusing on the kidney. Friedli et al. suggested that △ADC (the cortico-medullary ADC difference) of RS-DWI had a better correlation with fibrosis than conventional DWI in patients with chronic kidney disease^19^. He et al. considered Z-DWI to have better image quality, less distortion and susceptibility artifacts than the conventional DWI^13^. Tavakoli et al. found that simultaneous multislice RT- DWI of the kidney reduces scan acquisition time by 30% and yields substantially improved image quality to enable better lesion characterization than FB-DWI^20^. However, the studies compared only two different DWI sequences and did not systematically compare the ADC reliability, c-mCNR and image quality of the commonly used renal DWI sequences. Furthermore, most studies drew regions of interest (ROI) in only one part of the kidney and did not separate ROIs for the renal cortex and medulla when taking the ADC measurements.

Therefore, the aim of our study is to systematically compare the reliability of ADC measurement in the renal cortex and medulla, c-mCNR and image quality among BH-DWI, FB-DWI, NT-DWI, RT-DWI, RS-DWI and Z-DWI, and then obtain the optimal renal DWI, which can be recommended for clinical application.

## Results

### Intra- and interobserver agreement of ADC measurements with the six techniques in kidney

The average ADC values of the two representative anatomic sites (cortex and medulla) were obtained for both kidneys, with the lowest values obtained using Z-DWI (Table 1) for reader 1 and reader 2. The ICC of ADC measurements in the cortex were higher than that in the medulla for the six DWI sequences. For example, the range of ADC values was 1.385–2.116 × 10^−3^ mm^2^/s in cortex and 1.174–1.817 × 10^−3^ mm^2^/s in medulla for the first measurement of reader 1(Table 1). Z-DWI has a superior inter-observer agreement of ADC measurements in the cortex and medulla in each kidney (LKC: 0.856, LKM: 0.798, RKC: 0.855, RKM: 0.808, all *P* > 0.05) than BH-DWI, FB-DWI, NT-DWI, RS-DWI, and RT-DWI (some *P* < 0.05). For example, in the first measurements of the two readers, the average ADC values of Z-DWI for the LKM ((1.240 ± 0.026) × 10^−3^ mm^2^/s vs. (1.264 ± 0.021) × 10^−3^ mm^2^/s; *P* = 0.238) had less variation compared with RS-DWI ((1.749 ± 0.034) × 10^−3^ mm^2^/s vs. (1.679 ± 0.028) × 10^−3^ mm^2^/s; *P* < 0.003). In addition, Z-DWI yielded the highest intra-observer ICCs (0.876–0.944, all *P* > 0.05) among the six DWI sequences (Table 1). For example, in reader 1’s two measurements with Z-DWI, the average ADC values for the LKC ((1.463 ± 0.027) × 10^−3^ mm^2^/s vs. (1.472 ± 0.025) × 10^−3^ mm^2^/s; *P* = 0.512) had less variation compared with RT-DWI ((1.896 ± 0.020) × 10^−3^ mm^2^/s vs. (1.964 ± 0.019) × 10^−3^ mm^2^/s; *P* < 0.001).

**Table 1:** The ADC measurement in six DWI techniques and their Intra- and Interobserver agreement between them.

			ADCs measured by Reader 1					ADCs measured by Reader 2			ADC agreement	
			First	CV	Second	CV	*P* Value	First	CV	*P* Value	Intraobserver	Interobserver
BH-DWI	LK	Cortex	1.914 ± 0.024 (1.867–1.961)	0.012	1.917 ± 0.027 (1.863–1.971)	0.014	0.897	1.925 ± 0.026 (1.872–1.977)	0.014	0.641	0.802	0.736
		Medulla	1.583 ± 0.021 (1.541–1.626)	0.013	1.599 ± 0.023 (1.554–1.644)	0.014	0.374	1.568 ± 0.023 (1.522–1.613)	0.015	0.519	0.800	0.539
	RK	Cortex	1.905 ± 0.022 (1.862–1.948)	0.011	1.888 ± 0.023 (1.841–1.934)	0.012	0.437	1.941 ± 0.028 (1.886–1.996)	0.014	0.084	0.707	0.788
		Medulla	1.583 ± 0.019 (1.545–1.622)	0.012	1.576 ± 0.020 (1.536–1.616)	0.013	0.646	1.555 ± 0.020 (1.515–1.595)	0.013	0.230	0.817	0.450
FB-DWI	LK	Cortex	1.902 ± 0.020 (1.862–1.942)	0.010	1.878 ± 0.022 (1.835–1.922)	0.012	0.310	1.889 ± 0.021 (1.846–1.931)	0.011	0.500	0.556	0.700
		Medulla	1.631 ± 0.019 (1.593–1.668)	0.012	1.634 ± 0.019 (1.596–1672)	0.012	0.850	1.606 ± 0.020 (1.567–1.645)	0.012	0.205	0.750	0.655
	RK	Cortex	1.886 ± 0.017 (1.851–1.920)	0.009	1.874 ± 0.024 (1.827–1.922)	0.013	0.604	1.888 ± 0.027 (1.833–1.942)	0.014	0.936	0.628	0.574
		Medulla	1.647 ± 0.019 (1.609–1.685)	0.011	1.629 ± 0.020 (1.589–1.669)	0.012	0.277	1.627 ± 0.018 (1.592–1.662)	0.011	0.271	0.782	0.663
NT-DWI	LK	Cortex	1.886 ± 0.019 (1.849–1.923)	0.010	1.938 ± 0.017 (1.904–1.972)	0.009	0.004*	1.925 ± 0.018 (1.889–1.961)	0.009	0.013*	0.685	0.788
		Medulla	1.676 ± 0.019 (1.638–1.714)	0.011	1.689 ± 0.017 (1.654–1.723)	0.010	0.385	1.668 ± 0.018 (1.633–1.703)	0.011	0.510	0.816	0.874
	RK	Cortex	1.889 ± 0.020 (1.849–1.928)	0.010	1.903 ± 0.021 (1.862–1.944)	0.011	0.466	1.914 ± 0.021 (1.873–1.956)	0.011	0.099	0.707	0.838
		Medulla	1.668 ± 0.017 (1.635–1.701)	0.010	1.689 ± 0.019 (1.652–1.727)	0.011	0.136	1.690 ± 0.015 (1.659–1.721)	0.009	0.145	0.811	0.728
RS-DWI	LK	Cortex	2.058 ± 0.030 (2.000–2.116)	0.014	2.080 ± 0.025 (2.029–2.130)	0.012	0.413	2.059 ± 0.033 (1.994–2.125)	0.016	0.955	0.696	0.743
		Medulla	1.749 ± 0.034 (1.682–1.817)	0.019	1.701 ± 0.030 (1.642–1.760)	0.017	0.026*	1.679 ± 0.028 (1.623–1.734)	0.017	0.003*	0.872	0.837
	RK	Cortex	2.050 ± 0.030 (1.991–2.110)	0.014	2.025 ± 0.028 (1.969–2.080)	0.014	0.278	2.082 ± 0.029 (2.024–2.141)	0.014	0.180	0.788	0.813
		Medulla	1.730 ± 0.031 (1.667–1.792)	0.018	1.672 ± 0.026 (1.620–1.725)	0.016	0.016*	1.746 ± 0.030 (1.686–1.806)	0.017	0.503	0.806	0.817
RT-DWI	LK	Cortex	1.896 ± 0.020 (1.857–1.935)	0.010	1.964 ± 0.019 (1.926–2.001)	0.009	< 0.001*	1.914 ± 0.022 (1.870–1.958)	0.011	0.326	0.707	0.750
		Medulla	1.679 ± 0.020 (1.639–1.718)	0.012	1.709 ± 0.020 (1.668–1.749)	0.012	0.121	1.662 ± 0.017 (1.629–1.695)	0.010	0.379	0.707	0.646
	RK	Cortex	1.891 ± 0.023 (1.845–1.936)	0.012	1.952 ± 0.021 (1.910–1.994)	0.011	0.003*	1.891 ± 0.026 (1.839–1.942)	0.014	0.995	0.758	0.881
		Medulla	1.668 ± 0.022 (1.623–1.713)	0.013	1.701 ± 0.023 (1.656–1.746)	0.013	0.044*	1.696 ± 0.017 (1.663–1.729)	0.010	0.101	0.853	0.783
Z-DWI	LK	Cortex	1.463 ± 0.027 (1.410–1.517)	0.018	1.472 ± 0.025 (1.421–1.522)	0.017	0.512	1.509 ± 0.025 (1.489–1.549)	0.016	0.068	0.939	0.856
		Medulla	1.240 ± 0.026 (1.187–1.293)	0.021	1.235 ± 0.025 (1.185–1.286)	0.021	0.686	1.264 ± 0.021 (1.221–1.306)	0.017	0.238	0.944	0.798
	RK	Cortex	1.429 ± 0.022 (1.385–1.472)	0.015	1.431 ± 0.024 (1.384–1.478)	0.016	0.848	1.459 ± 0.024 (1.411–1.508)	0.017	0.066	0.876	0.855
		Medulla	1.220 ± 0.023 (1.174–1.266)	0.019	1.211 ± 0.023 (1.165–1.257)	0.019	0.507	1.256 ± 0.022 (1.211–1.300)	0.018	0.053	0.916	0.808

ADC are given in *10^−3^mm^2^/s. Mean ADC values measured in different anatomical regions. Data in parentheses are 95% confidence intervals. *P* values were gained by using the paired t test to compare differences for reader1 between the first and second ADC measurement, and to compare for the first ADC measurement between reader1 and reader2. *P* < 0.05 were considered significant difference (*).

*LK* left kidney, *RK* right kidney, *CV* coefficient of variation, *BH-DWI* breath-hold DWI, *FB-DWI* free-breathing DWI, *NT-DWI* navigator-triggered DWI, *RS-DWI* readout-segmented DWI, *RT-DWI* respiratory-triggered DWI and *Z-DWI* Zoomit DWI.

### ADC reproducibility in the 12 anatomic locations with each technique

**Figure 1:**
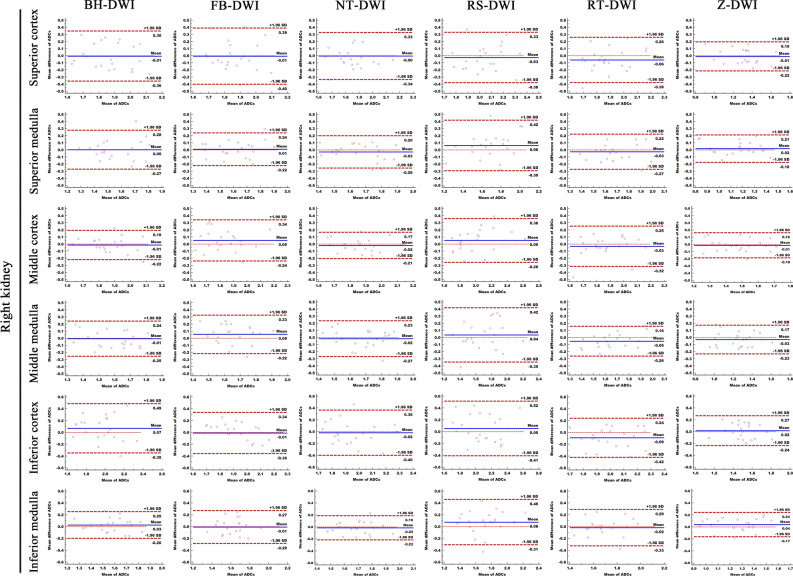
Comparison of ADC measurement repeatability of the six different DWI sequences in right kidney. The Bland–Altman plots of ADC measurements presented that Z-DWI had the lowest lowest LOA (0.033–0.056 × 10^−3^ mm^2^/s) (near zero) than all other sequences at almost all measurement points (6 anatomic locations of right kidney), which indicates that Z-DWI has the best ADC measurement repeatability.

**Figure 2:**
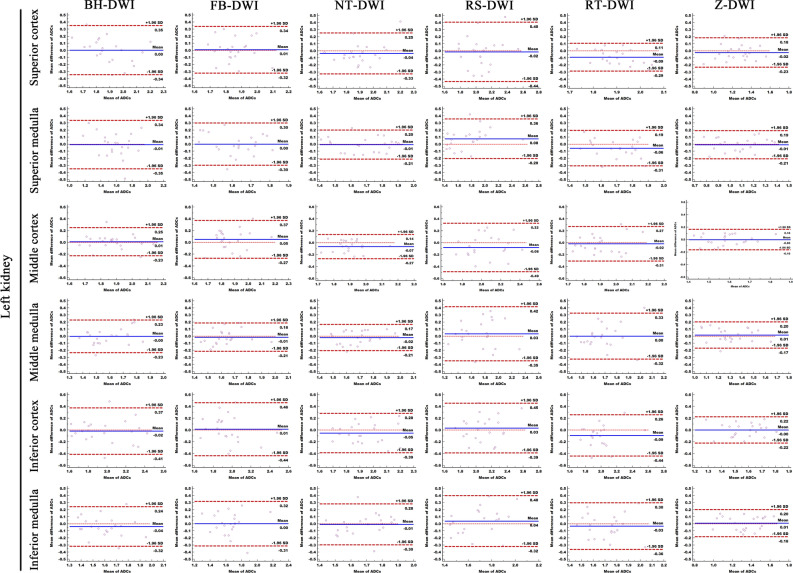
Comparison of ADC measurement repeatability of the six different DWI sequences in left kidney. The Bland–Altman plots of ADC measurements presented that Z-DWI had the lowest lowest LOA (0.031–0.051 × 10^−3^ mm^2^/s) (near zero) than all other sequences at almost all measurement points (6 anatomic locations of left kidney), which indicates that Z-DWI has the best ADC measurement repeatability.

All coefficient of variation (CV) fell between 0.9 and 2.1% in reader1 and reader 2. The repeatability of ADC measurements in the 12 anatomic locations varied for each technique. The mean ADC absolute differences (bias) with Z-DWI was 0.070–0.111 × 10^−3^ mm^2^/s, which was lower than BH-DWI (0.083–0.181 × 10^−3^ mm^2^/s), FB-DWI (0.087–0.186 × 10^−3^ mm^2^/s), NT-DWI (0.076–0.150 × 10^−3^ mm^2^/s), RS-DWI (0.125–0.203 × 10^−3^ mm^2^/s) and RT-DWI (0.096–0.148 × 10^−3^ mm^2^/s) for the 12 representative locations. Furthermore, Z-DWI had the highest ADC measurement repeatability, with the lowest LOA (0.031–0.056 × 10^−3^ mm^2^/s) than all other sequences (Table 2, Figs. 1, 2 and Supplementary Table 1).

**Table 2:** The mean absolute differences of ADCs measurement and their 95% confidence interval in twelve anatomic locations with six DWI techniques.

Locations		BH-DWI	FB-DWI	NT-DWI	RS-DWI	RT-DWI	Z-DWI
Cortex	Right superior (R1)	0.158 (0.070)	0.155 (0.111)	0.135 (0.087)	0.148 (0.092)	0.134 (0.094)	0.096 (0.033)
	Right middle (R3)	0.083 (0.056)	0.121 (0.085)	0.076 (0.051)	0.134 (0.083)	0.098 (0.098)	0.071 (0.036)
	Right inferior (R5)	0.181 (0.112)	0.144 (0.089)	0.149 (0.108)	0.203 (0.111)	0.148 (0.106)	0.111 (0.056)
	Left superior (L1)	0.137 (0.095)	0.131 (0.091)	0.114 (0.087)	0.157 (0.127)	0.111 (0.068)	0.090 (0.050)
	Left middle (L3)	0.087 (0.074)	0.139 (0.086)	0.097 (0.066)	0.180 (0.112)	0.125 (0.069)	0.070 (0.045)
	Left inferior (L5)	0.147 (0.119)	0.186 (0.114)	0.150 (0.083)	0.176 (0.110)	0.145 (0.123)	0.097 (0.051)
Medulla	Right superior (R2)	0.103 (0.082)	0.091 (0.063)	0.090 (0.068)	0.148 (0.105)	0.100 (0.070)	0.077 (0.055)
	Right middle (R4)	0.111 (0.050)	0.125 (0.068)	0.102 (0.068)	0.154 (0.108)	0.096 (0.061)	0.090 (0.049)
	Right Inferior (R6)	0.096 (0.059)	0.116 (0.069)	0.082 (0.055)	0.165 (0.110)	0.113 (0.096)	0.091 (0.054)
	Left superior (L2)	0.133 (0.098)	0.119 (0.081)	0.084 (0.054)	0.125 (0.089)	0.107 (0.078)	0.084 (0.048)
	Left middle (L4)	0.089 (0.066)	0.087 (0.046)	0.081 (0.045)	0.157 (0.104)	0.121 (0.098)	0.081 (0.043)
	Left inferior (L6)	0.115 (0.080)	0.120 (0.092)	0.105 (0.091)	0.148 (0.099)	0.136 (0.089)	0.090 (0.031)

The mean absolute differences of ADC Measurement were given in *10^−3^mm^2^/s, which were calculated between the first and second DW imaging series and the differences represented ADC reproducibility. The 95%confidence interval of the mean absolute differences (limits of agreement [LOAs]) were shown in the parentheses.

*BH-DWI* breath-hold DWI, *FB-DWI* free-breathing DWI, *NT-DWI* navigator-triggered DWI, *RS-DWI* readout-segmented DWI, *RT-DWI* respiratory- triggered DWI and *Z-DWI* Zoomit DWI.

### Measurement of cortico-medullary contrast to noise ratio(c-mCNR)

For the measurement of c-mCNR, a good agreement between reader 1 and reader 2 was found in the upper pole (RK: r = 0.779; LK: r = 0.891), middle pole (RK: r = 0.775; LK: r = 0.818) and lower pole (RK: r = 0.72; LK: r = 0.854) with Z-DWI (Tables 3 and 4). Furthermore, the Z-DWI has a slightly higher c-mCNR than other DWIs in most representative locations (*P* > 0.05). Notably, it is significant higher than BH-DWI and FB-DWI in the middle pole of bilateral kidney and the upper pole of the left kidney (*P* < 0.05). For example, in the middle pole of the right kidney, the c-mCNR was 12.62 ± 3.02 (95% CI: 11.29–13.96) with Z-DWI measured by reader 1, which was slightly higher than that with RS-DWI (9.70 ± 6.00 (95% CI: 7.04–12.36), *P* > 0.05), NT-DWI (9.64 ± 3.48 (95% CI: 8.10–11.19), *P* > 0.05), and RT-DWI (9.70 ± 6.00 (95% CI: 7.04–12.36), *P* > 0.05), but significantly higher than that with FB-DWI (7.14 ± 2.94 (95% CI: 5.84–8.44), *P* < 0.001) and BH-DWI (8.62 ± 6.14 (95%CI: 5.89–11.34), *P* = 0.004) (Tables 3 and 4).

**Table 3:** Cortico-medullary contrast to noise ratio (c-mCNR) of right kidney.

Sequence	The upper pole			The middle pole			The lower pole		
	Reader 1	Reader 2	Agreement	Reader 1	Reader 2	Agreement	Reader 1	Reader 2	Agreement
BH-DWI (1)	8.16 ± 6.33 (5.35–10.97)	8.58 ± 4.57 (6.55–10.60)	0.236	8.62 ± 6.14 (5.89–11.34)	7.19 ± 4.14 (5.36–9.03)	0.609	13.04 ± 8.71 (9.18–16.90)	14.64 ± 7.02 (11.53–17.75)	0.357
FB-DWI (2)	5.88 ± 3.10 (4.51–7.26)	6.26 ± 3.37 (4.76–7.75)	0.855	7.14 ± 2.94 (5.84–8.44)	7.31 ± 3.18 (5.90–8.72)	0.868	13.67 ± 7.54 (10.32–17.01)	15.74 ± 8.59 (11.93–19.55)	0.645
NT-DWI (3)	9.77 ± 4.44 (7.80–11.74)	9.63 ± 3.74 (7.98–11.29)	0.785	9.64 ± 3.48 (8.10–11.19)	9.66 ± 3.56 (8.08–11.24)	0.829	14.75 ± 6.10 (12.05–17.46)	14.63 ± 6.01 (11.96–17.29)	0.737
RS-DWI (4)	10.10 ± 5.75 (7.55–12.65)	10.71 ± 6.10 (8.01–13.41)	0.567	11.21 ± 5.72 (8.67–13.74)	12.86 ± 6.69 (9.89–15.83)	0.673	14.54 ± 4.78 (12.42–16.66)	14.33 ± 7.05 (11.21–17.46)	0.577
RT-DWI (5)	7.37 ± 4.58 (5.34–9.40)	7.31 ± 3.61 (5.71–8.91)	0.854	9.70 ± 6.00 (7.04–12.36)	10.44 ± 5.62 (7.95–12.93)	0.69	14.91 ± 8.34 (11.21–18.61)	13.20 ± 6.84 (10.17–16.24)	0.808
Z-DWI (6)	9.57 ± 3.06 (8.21–10.93)	11.10 ± 4.46 (9.12–13.07)	0.779	12.62 ± 3.02 (11.29–13.96)	13.22 ± 3.22 (11.79–14.64)	0.775	14.44 ± 4.26 (12.55–16.33)	15.18 ± 3.66 (13.55–16.80)	0.72
*P*-value for comparisons in Reader1									
Overall	0.004*			< 0.001*			0.509		
Pairwise	2–3: 0.008			1–6: 0.004					
	2–4: 0.033			2–6 < 0.001					

*P* < 0.05 were considered significant difference (*). *P* values were presented only when they were less than 0.05. c-mCNR (Cortico-medullary contrast to noise ratio) was measured in different anatomical regions with b = 800 s/mm^2^ images. Mean values of c-mCNR with the standard deviation and 95% confidence interval in parentheses are shown. At the bottom, *P*-values for the overall comparisons using Friedman test are given in reader1. If the Friedman test found a statistically significant *P*-value, additional *P*-value was presented with all pairwise comparisons by the Dunn-Bonferroni post-hoc test.

*BH-DWI* breath-hold DWI, *FB-DWI* free-breathing DWI, *NT-DWI* navigator-triggered DWI, *RS-DWI* readout-segmented DWI, *RT-DWI* respiratory-triggered DWI and *Z-DWI* Zoomit DWI.

**Table 4:** Cortico-medullary contrast to noise ratio (c-mCNR) of left kidney.

Sequence	The upper pole			The middle pole			The lower pole		
	Reader 1	Reader 2	Agreement	Reader 1	Reader 2	Agreement	Reader 1	Reader 2	Agreement
BH-DWI (1)	6.33 ± 5.99 (3.68–8.99)	7.18 ± 5.22 (4.86–9.49)	0.474	9.92 ± 5.55 (7.46–12.39)	11.01 ± 5.22 (8.70–13.33)	0.633	13.69 ± 7.13 (10.53–16.85)	14.94 ± 7.27 (11.72–18.16)	0.87
FB-DWI (2)	4.32 ± 3.16 (2.91–5.72)	5.79 ± 3.51 (4.23–7.35)	0.362	11.25 ± 4.81 (9.12–13.38)	12.00 ± 4.17 (10.14–13.85)	0.816	12.25 ± 8.14 (8.64–15.86)	14.80 ± 7.91 (11.29–18.30)	0.64
NT-DWI (3)	8.32 ± 3.75 (6.65–9.98)	8.32 ± 3.61 (6.72–9.92)	0.703	11.03 ± 4.23 (9.16–12.91)	10.87 ± 4.41 (8.91–12.82)	0.901	16.75 ± 8.84 (12.83–20.67)	17.32 ± 10.09 (12.84–21.79)	0.85
RS-DWI (4)	8.43 ± 4.55 (6.42–10.45)	10.31 ± 5.49 (7.87–12.74)	0.499	12.31 ± 5.04 (10.08–14.54)	15.38 ± 5.79 (12.81–17.95)	0.499	14.81 ± 7.69 (11.40–18.22)	15.57 ± 6.32 (12.77–18.38)	0.783
RT-DWI (5)	8.28 ± 3.73 (6.62–9.93)	8.44 ± 3.53 (6.88–10.01)	0.847	11.83 ± 6.28 (9.04–14.61)	14.53 ± 7.97 (11.00–18.07)	0.839	14.19 ± 7.83 (10.72–17.66)	15.06 ± 7.02 (11.95–18.17)	0.89
Z-DWI (6)	9.46 ± 3.01 (8.13–10.80)	10.45 ± 3.27 (9.00–11.90)	0.891	15.93 ± 5.87 (13.33–18.54)	16.74 ± 3.79 (15.06–18.42)	0.818	16.02 ± 3.79 (14.34–17.70)	18.23 ± 4.10 (16.41–20.05)	0.854
**P-value for comparisons in Reader1**									
Overall	< 0.001*			0.003*			0.255		
Pairwise	1–6: 0.043			1–6: 0.001					
	2–6 < 0.001			2–6: 0.033					
	2–3: 0.004								
	2–4: 0.001								
	2–5: 0.006								

*P* < 0.05 were considered significant difference (*). *P* values were presented only when they were less than 0.05. c-mCNR (Cortico-medullary contrast to noise ratio) was measured in different anatomical regions with b = 800 s/mm^2^ images. Mean values of c-mCNR with the standard deviation and 95% confidence interval in parentheses are shown. At the bottom, *P*-values for the overall comparisons using Friedman test are given in reader1. If the Friedman test found a statistically significant *P*-value, additional *P*-value was presented with all pairwise comparisons by the Dunn-Bonferroni post-hoc test.

*BH-DWI* breath-hold DWI, *FB-DWI* free-breathing DWI, *NT-DWI* navigator-triggered DWI, *RS-DWI* readout-segmented DWI, *RT-DWI* respiratory- triggered DWI and *Z-DWI* Zoomit DWI.

### Image quality analysis

**Figure 3:**
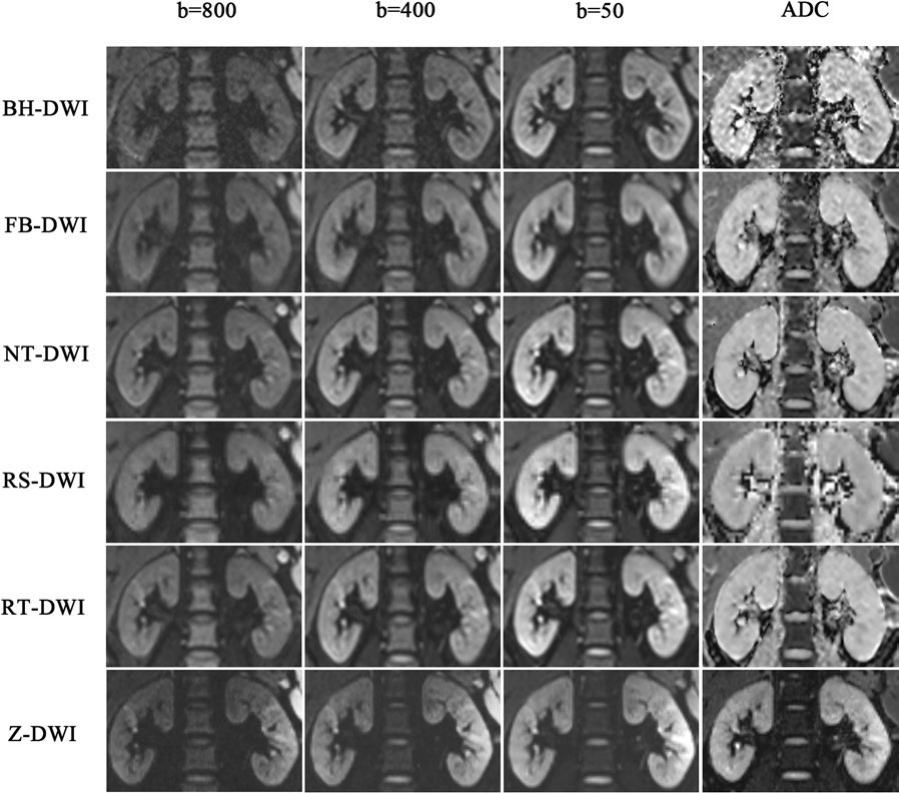
Comparisons of image quality of BH-DWI, FB-DWI, NT-DWI, RT-DWI, RS-DWI and Z-DWI. Diffusion-weighted trace images at three different b-values (800, 400, 50 s/mm^2^) with the corresponding ADC maps (right) are arrayed. Z-DWI had a better image quality than BH-DWI and FB-DWI in clarity of the renal cortex and medulla (K4) (ADC map) (all *P* < 0.05) and RS-DWI in sharpness of boundaries (K1), clarity of the renal cortex and medulla (K4) and overall image quality(K5) (all *P* < 0.05). Z- DWI was slightly superior to RT- DWI and NT-DWI in sharpness of boundaries (K1, ADC map); however, the difference in image quality between the three was not significant (all *P* > 0.05).

**Figure 4:**
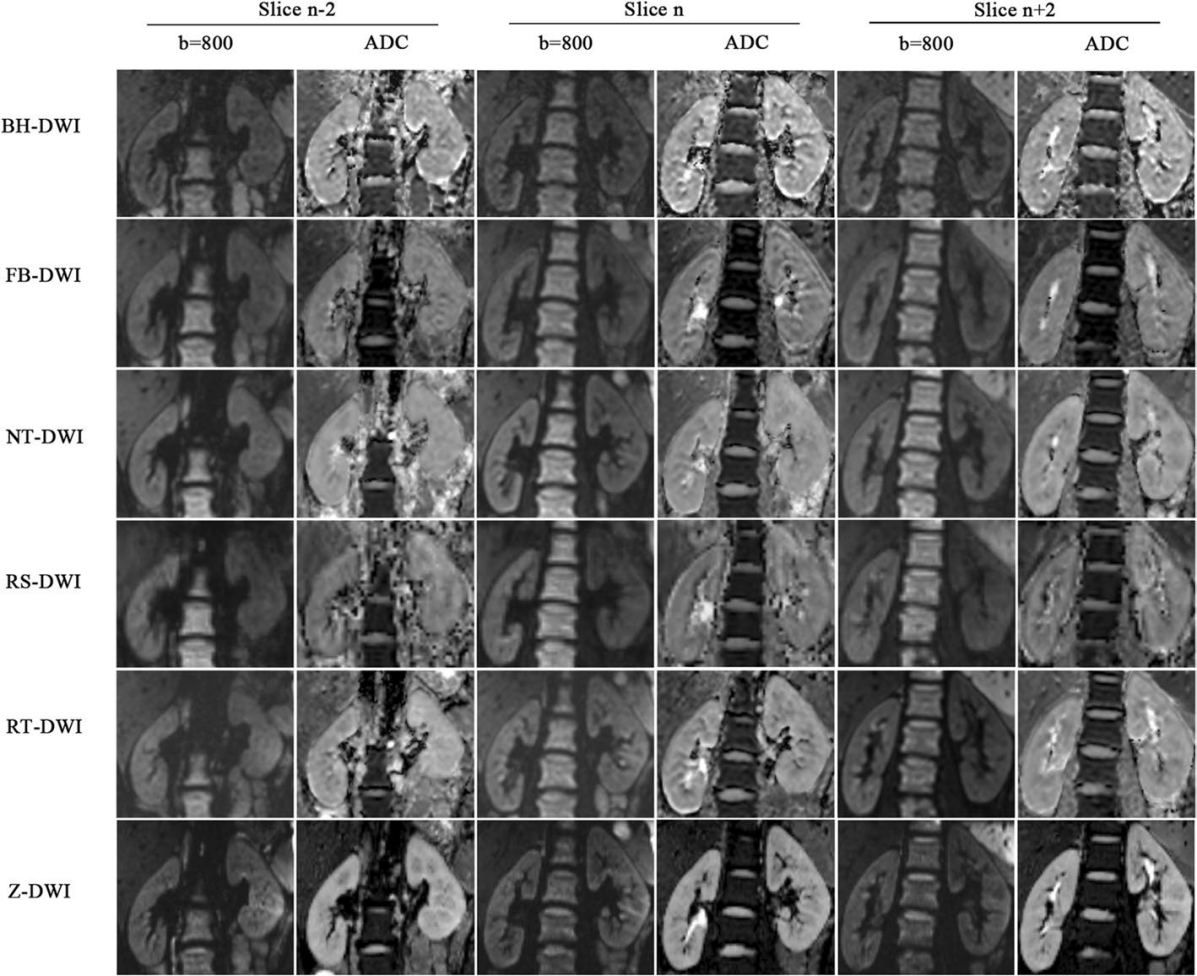
Comparisons of image quality of BH-DWI, FB-DWI, NT-DWI, RT-DWI, RS-DWI and Z-DWI at b = 800 s/mm^2^ and corresponding ADC map. The image quality of Z-DWI was significantly different from BH-DWI, FB-DWI and RS-DWI in the three representative section (n-2 slice, n slice and n + 2 slice) in clarity of the renal cortex and medulla (ADC map) (all *P* < 0.05). Z- DWI was slightly superior to RT- DWI and NT-DWI in the three representative section (n-2 slice, n slice and n + 2 slice) in sharpness of boundaries (ADC map); however, the difference in image quality between the three was also not significant (all *P* > 0.05).

The two readers had an excellent agreement in evaluating the five aspects (K1-K5) of image quality (Kappa value 0.945–0.989). Z-DWI had a high score in terms of image blurring (5 points), severity of artifacts (4 points), sharpness of boundaries (5 points), clarity of the renal cortex and medulla (5 points), and overall image quality (5 points), which was similar with the image quality of RT-DWI and NT-DWI (*P* > 0.05). However, Z-DWI had a better image quality than BH-DWI in K4 **(**ADC map) (*P* < 0.05), FB-DWI in K2 (all *P* < 0.05), K4 and K5 (ADC map) (all *P* < 0.05), and RS-DWI in all image quality aspects except for K2 and K4 (ADC map) (all *P* < 0.05) (Table 5, Figs. 3 and 4).

**Table 5:** The evaluation of image quality in volunteer by reader 3(R3) and reader 4(R4).

Criteria	BH-DWI (1)		FB-DWI (2)		NT-DWI (3)		RS-DWI (4)		RT-DWI (5)		Z-DWI (6)		P values for comparisons		Kappa value
	R3	R4	R3	R4	R3	R4	R3	R4	R3	R4	R3	R4	Overall	Pairwise	
**K1: Imaging blur**															0.966 (*P* < 0.0001)
ADC map	5 (4,5)	5 (4,5)	4 (2,5)	4 (2,5)	5 (4,5)	5 (4,5)	4 (2,5)	4 (2,5)	5 (3,5)	5 (3,5)	5 (4,5)	5 (4,5)	< 0.0001	6–4: *P* < 0.001; 4–1/3/5: *P* < 0.001	
B = 50	5 (4,5)	5 (4,5)	4 (2,5)	4 (2,5)	5 (4,5)	5 (4,5)	4 (3,4)	4 (2,4)	5 (4,5)	5 (4,5)	5 (4,5)	5 (4,5)	< 0.0001	6–4: *P* < 0.001; 2–1: *P* = 0.033; 2–5: *P* = 0.043; 4–1/3/5: *P* < 0.001	
B = 400	5 (4,5)	5 (4,5)	4 (2,5)	4 (2,5)	5 (4,5)	5 (4,5)	4 (3,5)	4 (2,5)	5 (4,5)	5 (4,5)	5 (4,5)	5 (4,5)	< 0.0001	6–4: *P* < 0.001; 2–1: *P* = 0.033; 2–5: *P* = 0.043; 4–1/3/5: *P* < 0.001	
B = 800	5 (4,5)	5 (4,5)	4 (2,5)	4 (2,5)	5 (4,5)	5 (4,5)	4 (3,5)	4 (2,5)	5 (4,5)	5 (4,5)	5 (4,5)	5 (4,5)	< 0.0001	6–4: *P* < 0.001; 2–1: *P* = 0.033; 2–5: *P* = 0.043; 4–1/3/5: *P* < 0.001	
**K2: severity of artifacts**															0.953 (*P* < 0.0001)
ADC map	5 (4,5)	5 (4,5)	4 (2,5)	4 (2,5)	5 (4,5)	5 (4,5)	4 (3,4)	4 (3,4)	5 (3,5)	5 (3,5)	4 (4,5)	4 (4,5)	< 0.0001	6–2: *P* = 0.049; 6–4: *P* = 0.009; 2–1/5: *P* < 0.001; 2–3: *P* = 0.001; 4–1/3/5: *P* < 0.001	
B = 50	5 (4,5)	5 (4,5)	4 (3,5)	4 (3,5)	5 (4,5)	5 (4,5)	4 (3,4)	4 (3,4)	5 (3,5)	5 (3,5)	4 (4,5)	4 (4,5)	< 0.0001	6–2: *P* = 0.049; 6–4: *P* = 0.009; 2–1/5: *P* < 0.001; 2–3: *P* = 0.001; 4–1/3/5: *P* < 0.001	
B = 400	5 (4,5)	5 (4,5)	4 (3,5)	4 (3,5)	5 (4,5)	5 (4,5)	4 (3,4)	4 (3,4)	5 (3,5)	5 (3,5)	4 (4,5)	4 (4,5)	< 0.0001	6–2: *P* = 0.049; 6–4: *P* = 0.009; 2–1/5: *P* < 0.001; 2–3: *P* = 0.001; 4–1/3/5: *P* < 0.001	
B = 800	5 (4,5)	5 (4,5)	4 (3,5)	4 (3,5)	5 (4,5)	5 (4,5)	4 (3,4)	4 (3,4)	5 (3,5)	5 (3,5)	4 (4,5)	4 (4,5)	< 0.0001	6–2: *P* = 0.049; 6–4: *P* = 0.009; 2–1/5: *P* < 0.001; 2–3: *P* = 0.001; 4–1/3/5: *P* < 0.001	
**K3: sharpness of boundaries**															0.989 (*P* < 0.0001)
ADC map	5 (5,5)	5 (5,5)	5 (2,5)	5 (2,5)	5 (4,5)	5 (4,5)	4 (3,5)	4 (3,5)	5 (4,5)	5 (4,5)	5 (4,5)	5 (4,5)	< 0.0001	6–4: *P* < 0.001; 4–2: *P* = 0.022; 4–5: *P* = 0.001; 4–1/3: *P* < 0.001	
B = 50	5 (5,5)	5 (5,5)	5 (2,5)	5 (2,5)	5 (4,5)	5 (4,5)	4 (3,5)	4 (3,5)	5 (4,5)	5 (4,5)	5 (4,5)	5 (4,5)	< 0.0001	6–4: *P* < 0.001; 4–2: *P* = 0.033; 4–1/3/5: *P* = 0.001	
B = 400	5 (5,5)	5 (5,5)	5 (2,5)	5 (2,5)	5 (4,5)	5 (4,5)	4 (3,5)	4 (3,5)	5 (4,5)	5 (4,5)	5 (4,5)	5 (4,5)	< 0.0001	6–4: *P* < 0.001; 4–2: *P* = 0.038; 4–1/5: *P* = 0.001; 4–3: *P* < 0.001	
B = 800	5 (5,5)	5 (5,5)	5 (2,5)	5 (2,5)	5 (4,5)	5 (4,5)	4 (3,5)	4 (3,5)	5 (4,5)	5 (4,5)	5 (4,5)	5 (4,5)	< 0.0001	6–4: *P* < 0.001; 4–2: *P* = 0.038; 4–1/5: *P* = 0.001; 4–3: *P* < 0.001	
**K4: clarity of the renal cortex and medulla**															0.945 (*P* < 0.0001)
ADC map	4 (3,4)	4 (3,4)	4 (2,4)	4 (2,4)	4 (3,5)	4 (3,5)	3 (2,4)	3 (2,4)	4 (3,5)	4 (3,5)	5 (3,5)	5 (3,5)	< 0.0001	6–2: *P* = 0.001; 6–1/4: *P* < 0.001; 4–3/5: *P* < 0.001; 1–3: *P* = 0.029	
B = 50	5 (5,5)	5 (5,5)	5 (3,5)	5 (3,5)	5 (3,5)	5 (3,5)	4 (3,5)	4 (2,5)	5 (4,5)	5 (4,5)	5 (4,5)	5 (4,5)	< 0.0001	6–4: *P* = 0.001; 4–1/2/3/5: *P* < 0.001	
B = 400	5 (5,5)	5 (5,5)	5 (3,5)	5 (3,5)	5 (4,5)	5 (4,5)	4 (3,5)	4 (2,5)	5 (4,5)	5 (4,5)	5 (4,5)	5 (4,5)	< 0.0001	6–4: *P* = 0.001; 4–1/2/3/5: *P* < 0.001	
B = 800	5 (4,5)	5 (4,5)	5 (3,5)	5 (3,5)	5 (4,5)	5 (4,5)	4 (3,5)	4 (2,5)	5 (4,5)	5 (4,5)	5 (3,5)	5 (3,5)	< 0.0001	6–4: *P* < 0.001; 4–1: *P* = 0.002; 4–2/3/5: *P* < 0.001	
**K5: Overall image quality**															0.971 (*P* < 0.0001)
ADC map	4.5 (4,5)	4.5 (4,5)	4 (2,5)	4 (2,5)	5 (4,5)	5 (4,5)	4 (2,4)	4 (2,4)	5 (3,5)	5 (3,5)	5 (4,5)	5 (4,5)	< 0.0001	6–2: *P* = 0.022; 6–4: *P* < 0.001; 2–3: *P* = 0.017; 2–5: *P* = 0.017; 4–1: *P* = 0.012; 4–3/5: *P* < 0.001	
B = 50	5 (5,5)	5 (5,5)	4 (2,5)	4 (2,5)	5 (3,5)	5 (3,5)	4 (3,4)	4 (3,4)	5 (4,5)	5 (4,5)	5 (4,5)	5 (4,5)	< 0.0001	6–4: *P* < 0.001; 2–1: *P* = 0.008; 2–5: *P* = 0.038; 4–1/3/5: *P* < 0.001	
B = 300	5 (5,5)	5 (5,5)	4 (2,5)	4 (2,5)	5 (4,5)	5 (4,5)	4 (3,4)	4 (3,4)	5 (4,5)	5 (4,5)	5 (4,5)	5 (4,5)	< 0.0001	6–4: *P* < 0.001; 2–1: *P* = 0.007; 2–3: *P* = 0.029; 2–5: *P* = 0.038; 4–1/3/5: *P* < 0.001	
B = 800	5 (4,5)	5 (4,5)	4 (2,5)	4 (2,5)	5 (4,5)	5 (4,5)	4 (3,4)	4 (3,4)	5 (4,5)	5 (4,5)	5 (4,5)	5 (4,5)	< 0.0001	6–4: *P* < 0.001; 2–1: *P* = 0.014; 2–3: *P* = 0.029; 2–5: *P* = 0.038; 4–1/3/5: *P* < 0.001	

Median (min, max) values for the mean image quality ratings calculated are given separately for the three different b-values as well as for the ADC map. 5 = excellent, 4 = good, 3 = moderate, 2 = fair, 1 = nondiagnostic. On the right, *P*-values for the overall comparisons using Friedman test are given. If the Friedman test showed a statistically significant *P*-value, the Dunn-Bonferroni post-hoc test for all pairwise comparisons was performed. *P* < 0.05 were considered significant difference. *P* values were presented only when they were less than 0.05.

*BH-DWI* breath-hold DWI, *FB-DWI* free-breathing DWI, *NT-DWI* navigator-triggered DWI, *RS-DWI* readout-segmented DWI, *RT-DWI* respiratory- triggered DWI and *Z-DWI* Zoomit DWI.

## Discussion

Currently, BH-DWI, FB-DWI, NT-DWI, RS-DWI, RT-DWI and Z-DWI have widely been used for the diagnosis of renal diseases and evaluate renal function^3,6,8,12,13^. For these DWIs, the reliability of ADC value and good image quality are vital in detecting renal disease and assessing renal function accurately. To our knowledge, this is the first MRI study to compare these DWIs systematically, by evaluating the intra- and inter-observer agreement in ADC measurements, reproducibility of ADC values and image quality to establish the most reliable clinically applicable renal DWI sequence.

ADC values derived from coronal renal DWI exhibited moderate-to-good agreement to axial DWI^11^. In our study, coronal renal DWI was performed because it can provide full coverage of the kidney shape. The mean ADC value in the renal cortex falls between 1.429 and 2.082 × 10^−3^ mm^2^/s and 1.211–1.749 × 10^−3^ mm^2^/s in the medulla in 12 representative sections (the upper, middle, and lower pole of both kidneys), which were near the lower limit of the values reported in the literature ((1.78 ± 0.11) × 10^−3^ mm^2^/s for the renal cortex and (1.48 ± 0.13) × 10^−3^ mm^2^/s for renal medulla in healthy volunteers^21^. Furthermore, our results showed that the 95% CI of ADC measurements in the cortex was higher than that in the medulla using the six DWI sequences, consistent with Sulkowska et al.^22^. Previous ADC values obtained with NT-DWI^10^ and RS-DWI^23^ were similar to our findings but were slightly higher with BH-DWI^10^ and Z-DWI^13^ than that in our results. In our results, Z-DWI yielded lower ADC values in the cortex and the medulla than the other five DWIs, which is consistent with Cai et al. findings that showed that the mean tumor ADC values of rFOV-DWI were significantly lower than those of fFOV-DWI (1.237 ± 0.228 × 10^−3^ mm^2^/s vs 1.683 ± 0.322 × 10^−3^ mm^2^/s, *P* < 0.001) in patients with gastric cancer^24,25^. The possible reason was that DWI with reduced FOV produce images with sharper margins and anatomic structural visualization^26^, which is helpful in drawing ROI in renal the cortex and medulla, yielding a stable and low ADC value. This suggests that a lower ADC value should be used in clinical work when using Z-DWI.

In addition, Z-DWI has the best intra-observer agreement (intra-class ICCs: 0.906–0.944) and inter-observer agreement (inter-class ICCs: 0.798–0.856) among the six sequences, indicating that Z-DWI is sufficiently reliable and repeatable when assessing ADC measurements. The possible reason for this result is that a reduced (“zoomed”) FOV in the phase-encoding direction decreases the influence of gastrointestinal peristalsis and respiratory motion artifacts on kidney images. In addition, RT-DWI and NT-DWI can reduce the influence of motion artifacts by respiratory- and navigator-triggered techniques. However, it is at the cost of rather long and uncertain scan times (more than 120 s in both sequences), which can markedly increase patients' discomfort and sensitivity to motion^27^. Consequently, the intra- and inter-observer agreements with RT-DWI and NT-DWI were lower than with Z-DWI. Previous studies have shown that Z-DWI has obvious advantages in cervical cancer^28^, thyroid micronodules^29^, cervical spinal cord^30^, etc. It enables clearer identification of lesions and reduction of image artifacts. Our research has further verified its value in kidney applications. Moreover, we found that the CV was less than 3% in all measurements, suggesting that the ADC measurements were reliable and consistent in all DWIs.

Our results indicate that Z-DWI has the best ADC repeatability because it yielded the least mean absolute differences of ADCs and LOAs in all the anatomical sections. This finding may be related to the “zoomed” technique in the direction of phase-encoding, which, when combined with dynamic, spatially selective RF pulses, further improved image quality in renal imaging considerably more than other DWIs^15,31^. According to previous studies of abdominal organs, different breathing schemes will affect the absolute ADC value^32^. The study of Yıldırım İO et al.^33^ found that compared with conventional DWI sequences, Z-DWI may be more effective in the diagnosis and monitoring of treatment and postoperative responses in patients with varicocele. Therefore, the good repeatability of Z-DWI helps us to evaluate the ADC value of renal disease quantitatively. Our study verifies that Z-DWI has the best consistency and reproducibility, which is of great significance to the future clinical applications of renal DWI sequences. Our results also showed that all the LOAs were around 20–30% of the mean ADC values. This is in line with previous studies that recommended at least a 30% change in ADC values when evaluating a lesion's response to treatment with the same DWI technique^6,23^.

Renal cortico-medullary ADC difference is an important marker for differentiating renal diseases. A good agreement was found with Z-DWI for assessing c-mCNR (ICC > 0.70) in all representative locations, indicating the reliability of Z-DWI in assessing ADC measurements of renal lesions. Furthermore, Z-DWI has a slightly higher c-mCNR than other DWIs in most representative locations (*P* > 0.05), and significantly higher c-mCNR than BH-DWI and FB-DWI in the middle pole of both kidneys and the upper pole of the left kidney (*P* < 0.05), which is consistent with previous reports^34,35^. This suggests that the Z-DWI may be a good sequence for depicting and differentiating renal diseases.

The DWIs with a long scan time (like Z-DWI, RS-DWI, RT-DWI and NT-DWI) can reduce the artifacts in DWI protocols, but this in turn can markedly increase patient’s discomfort and decrease image quality. In our study, Z-DWI yielded a high score in terms of imaging blurring, sharpness of boundaries, clarity of the renal cortex and medulla, and overall image quality, which has the similar image quality to RT-DWI and NT-DWI (*P* > 0.05) and superior to RS-DWI (*P* < 0.05). The possible reason is that the “zoomed” technique in the direction of phase-encoding, combined with dynamic, spatially selective RF pulses reduced susceptibility artifacts markedly and gained considerable image quality improvements in renal imaging^15,31^. Although RS-DWI can reduce T2 blurring and susceptibility effects^12^, its long acquisition time (226–379 s in our study) makes it prone to motion artifacts, reducing the image quality, especially for the mobile kidney. Furthermore, Z-DWI had a higher score than BH-DWI in clarity of the renal cortex and medulla (ADC map, *P* < 0.05) and RS-DWI in clarity of the renal cortex and medulla (all *P* < 0.05). This indicates that imaging with Z-DWI provides a clearer margin between the renal cortex and medulla and helps to locate the orientation of renal lesions and precisely measure ADC value in the cortex and medulla. In addition, Z-DWI was better than FB-DWI and RS-DWI in severity of artifacts (*P* < 0.05), which is similar to a previous study where FB-DWI and RS-DWI had more artifacts compared to Z-DWI^13,36^.

This study also has some limitations. First, the volunteers included in this study are all young with better breathing coordination, which is somewhat different from the clinical situation of patients with kidney disease. Secondly, this study was performed in normal kidneys, without any lesions, to ensure the same condition of the kidney to avoid the bias of ADC measurements due to inhomogeneity that lesions might cause. Finally, in order to evaluate the image quality of different kidney regions, this study uses a coronal scan, which increases the impact of respiratory motion artifacts on the image.

In summary, Z-DWI had an excellent intra-observer agreement and good inter-observer agreement among the six sequences. Furthermore, Z-DWI had the highest ADC repeatability and c-mCNR in most of the 12 locations of the kidneys observed. In addition, Z-DWI had a similar image quality with RT-DWI and NT-DWI and better image quality than BH-DWI, FB-DWI and RS-DWI (*P* < 0.05). Therefore, Z-DWI is the optimal renal DWI sequence that can be used as a reliable quantitative parameter and therapeutic biomarker for patients with renal disease and evaluation of renal function. Thus, it is recommended as the DWI sequence for clinical examination of the kidney due to its good image quality and reliable diagnostic confidence.

## Materials and methods

### Ethics statement and participants’ enrollment

This prospective study was approved by the research ethics committee of our institution (Xiangya Hospital, Central South University, China). The authors confirm all data has informed written informed consent obtained from each participant. All methods were performed in accordance with the relevant guidelines and regulations and strictly abide by the Declaration of Helsinki. 22 healthy young volunteers with similar age (juniors in a medical college, mean: 21 years, range: 20–22 years) were enrolled (12 males, 10 females).

In our study, the inclusion criteria included: (a) no history of albuminuria, hematuria and weight loss; (b) no history of any kidney surgery; (c) ability of the subject to hold his or her breath for up to 20 seconds. The exclusion criteria included: (a) contraindications to MR imaging; (b) history of any kidney disease and surgery.

### MR imaging protocol

Magnetic resonance examinations were performed on a 3.0 T system (MAGNETOM Prisma, Siemens Healthcare, Erlangen, Germany) with an 18-channel anterior surface body coil combined with 12 elements of a 32-channel spine coil. Each subject was scanned twice in the DWI series. The DWI series included end-expiratory breath-hold DWI (BH-DWI) (one breath-hold), free-breathing DWI (FB-DWI), navigator-triggered DWI (NT-DWI), readout-segmented DWI (RS-DWI)(with respiratory-triggering), respiratory-triggered DWI (RT-DWI) and Zoomit DWI (Z-DWI) (with respiratory-triggering). Three b values of 50, 400, and 800 s/mm^2^ were sampled in three orthogonal diffusion directions (three-scan trace) for all DWIs. A 5 min rest was allowed between two identical sessions. The scan parameters were kept as close as possible, and the detailed parameters of all sequences are summarized in Table 6. The imaging parameters of the two scans were consistent. Each participant had 12 scans (six scans using the 6 techniques in each session). The fat suppression was achieved with spectral adiabatic inversion recovery in all DWI sequences, and the acceleration factors were 2 in all sequences. A k-space-based parallel imaging technique was used. The scan time was recorded.

**Table 6:** The summarized parameters of all DWI sequences in our study.

Parameters	BH-DWI	FB-DWI	NT-DWI	RS-DWI	RT-DWI	Z-DWI
Time of repetition (ms)	1500	3000	900	1200	1200	1800
Time of echo (ms)	55	55	55	54 (TE1)/91 (TE2)	55	71
Slices	11	18	18	18	18	15
Slice thickness (mm)	4.0	4.0	4.0	4.0	4.0	4.0
Slice gap (mm)	0	0	0	0	0	0
Bandwidth (Hz/Px)	2332	2332	2332	1435	2332	2222
Echo spacing (ms)	0.51	0.51	0.51	0.34	0.51	0.54
iPAT	GRAPPA 2	GRAPPA 2	GRAPPA 2	GRAPPA 2	GRAPPA 2	GRAPPA 2
Concatenations	1	1	3	3	3	2
Averages	1/1/1	2/2/4	2/2/4	1/2/2	2/2/4	2/2/4
b-values (s/mm^2^)	50,400,800	50,400,800	50,400,800	50,400,800	50,400,800	50,400,800
Coil elements	30 (18 body, 12 spine)	30 (18 body, 12 spine)	30 (18 body, 12 spine)	30 (18 body, 12 spine)	30 (18 body, 12 spine)	30 (18 body, 12 spine)
Field of view (mm)	400*240	400*240	400*240	400*240	400*240	300*184
Matrix	134*80	134*80	134*80	134*80	134*80	150*83
Voxel size(mm^3^)	3.0*3.0*4.0	3.0*3.0*4.0	3.0*3.0*4.0	3.0*3.0*4.0	3.0*3.0*4.0	2.0*2.2*4.0
Net scan time(s)	23	87	151	120	108	142
Range of scan time(s)	23	87	166–394	226–379	140–246	212–411
Mean of scan time(s)	23	87	301	306	186	314

*BH-DWI* breath-hold DWI, *FB-DWI* free-breathing DWI, *NT-DWI* navigator-triggered DWI, *RS-DWI* readout-segmented DWI, *RT-DWI* respiratory-triggered DWI, *Z-DWI* Zoomit DWI, *iPAT* integrated parallel acquisition technique, *GRAPPA* generalized autocalibrating partially parallel acquisition, *Concatenations* group acquisition.

### Image analysis

All the DWI images data were saved to the workstation. They were evaluated by four readers, including (1) ADC values, (2) the repeatability of ADC measurements, and (3) subjective image quality. The ADC values were calculated separately using the post-processing software (Syngo.via VB10, Siemens Healthcare). The measurements of ADC were done by two radiologists (WG.L. and H.L., readers 1 and 2, with 5 and 10 years of clinical imaging diagnosis experience, respectively), and two other radiologists assessed the subjective image quality (YG. P. and WZ. L., readers 3 and 4, with 15 and 20 years of clinical imaging diagnosis experience, respectively). Independent double-blinding was used in four readers throughout the measurement and evaluation process.

#### ADC value measurement and repeatability evaluation

**Figure 5:**
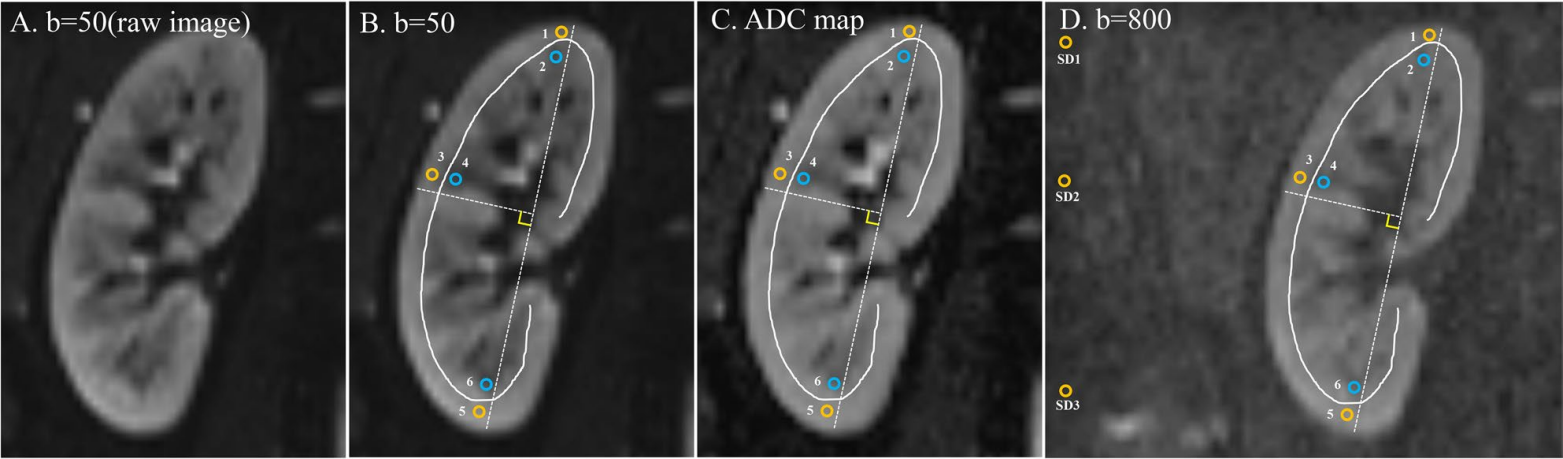
Schematic diagram of typical ROI placement in the renal cortex and medulla. Raw diffusion-weighted imaging (DWI) at b = 50 s/mm^2^ (**A**), six representative ROIs (3 ROIs each for the renal cortex and medulla in superior, middle and inferior zones, respectively) on DWI at b = 50 s/mm^2^ (**B**), corresponding ADC map (**C**) and b = 800 s/mm^2^ image (**D**) for c-mCNR measurements. First, the DWI slice (using b = 50 s/mm^2^) with the largest renal section was chosen and a straight line was drawn along the upper and lower poles of the kidney (white dashed line). Then, a perpendicular bisector was drawn (white dashed line). Second, the medullary zone adjacent to the white dashed, which has a clear lower signal intensity was identified and the ROIs representing the superior, middle and inferior zones were drawn manually. Subsequently, three similar ROIs were drawn in renal cortex based on the representative medulla positions. These ROIs were copied to the corresponding ADC map for ADC measurement. Moreover, the background signal standard deviation (SD) for c-mCNR measured using an equally sized ROI placed at a nearby background (air) in the corresponding section, close to the site of the kidney ROI, and avoiding any prominent artifacts.

12 ROIs were drawn on the b = 50 s/mm^2^ images, including the upper, middle and lower poles of cortex and medulla on both kidneys. ROIs 20-24mm^2^ in size^37,38^ were positioned the on the b = 50 s/mm^2^ image (Fig. 5A,B), and then copied to the ADC map for ADC measurements (Fig. 5C) and b = 800 s/mm^2^ images for c-mCNR measurements (Fig. 5D). Then, ROIs were drawn in the second scan and in the repeated series of the other five sequences in a similar manner. The ADC measurements were repeated one week after the first measurement to avoid recall bias. The second radiologist repeated the same measurement. The ADC value were gained using the following formula by the log-linear fitting algorithm with three different b factors (b = 50, 400, 800 s/mm^2^):

1 $$I_{Trace} = I_{0} e^{ - b*ADC} = I_{0} e^{{ - b*\frac{{\left( {D1 + D2 + D3} \right)}}{3}}} = \sqrt[3]{{I_{1} *I_{2} *I_{3} }}$$

I1, I2, and I3 are the measured diffusion-weighted images in three orthogonal gradient directions, and D1, D2 and D3 are the corresponding diffusion coefficients.

In addition, CV of ADC value was used to assess the relative degree of dispersion between ADC value measurements, which was calculated as the following formula:

2 $$CV_{ADC} = \frac{SD}{{ADC_{Mean} }} \times 100\%$$

Here, SD was the standard deviation of ADC value and ADC_Mean_ was the mean value of ADC value in various representive point^39^. It indicated that the ADC measurement was reliable when CV less than 0.15.

#### Cortico-medullary contrast to noise ratio (c-mCNR)

The signal intensity (SI) was measured in different anatomical regions with b = 800 s/mm^2^ images, including the upper, middle and lower poles of cortex and medulla on both kidneys. Moreover, the background signal standard deviation (SD) measured using an equally sized ROI placed at a nearby background (air) in the corresponding section, close to the site of the kidney ROI, and avoiding any prominent artifacts (Fig. 5D). The following formula was used to calculate the corresponding c-mCNR of different DWI sequences:

3 $$c - mCNR = \frac{{\left| {SI_{cortex} - SI_{medulla} } \right|}}{{SD_{background} }}$$

where SI_cortex_ and SI_medulla_ were the signal intensity of the specific position ROI (for instance, the upper pole of right kidney). SD_background_ was the standard deviation of the chosen artifact-free ROI positioned on the background (air) of the corresponding slice. In all volunteers, CNR were measured once in 1 week by reader 1 and reader 2. Mean values of c-mCNR with the standard deviation and 95% confidence interval were recorded.

#### The evaluation of image quality

The image quality of the six DWIs on the ADC map and DWI images at b = 50, 400 and 800 s/mm^2^ were evaluated by two radiologists (reader 3 and reader 4), respectively. The score criteria of image quality for each DWI are shown in Table 7.

**Table 7:** The criterion of the image quality scores for coronal kidney DWI in our study.

**Kidney (K1): imaging blur**	
1. Not Diagnostic	
2. Severe blurring	
3. Moderate blurring	
4. Mild blurring	
5. Sharp, no blurring	
**Kidney (K2): severity of artifacts**	
1. Not Diagnostic	
2. Severe artifacts	
3. Moderate artifacts	
4. Mild Artifacts	
5. No Artifacts	
**Kidney (K3): sharpness of boundaries**	
1. Not Diagnostic	
2. Poor, definitely affecting interpretation	
3. Moderate, potentially affecting interpretation	
4. Good, not affecting interpretation	
5. Excellent	
**Kidney (K4): clarity of the renal cortex and medulla**	
1. Not Diagnostic	
2. Poor, definitely affecting interpretation	
3. Moderate, potentially affecting interpretation	
4. Good, not affecting interpretation	
5. Excellent	
**Kidney (K5): overall image quality**	
1. Not Diagnostic	
2. Poor, definitely affecting interpretation	
3. Moderate, potentially affecting interpretation	
4. Good, not affecting interpretation	
5. Excellent	

### Statistical analysis

The mean value and standard deviation (SD) of ADC values of 12 ROIs in cortex and medulla on both kidneys were used to estimate the consistency of ADC measurement. The t-test was used to compare the difference between the first and second readers’ measurements (inter-observer agreement) and the difference between repeated measurements (intra-observer agreement). The intra- and inter-class correlation coefficients (ICCs) (and 95% confidence intervals) were used to evaluate the intra- and inter-observer agreement, respectively. An ICC value greater than 0.70 indicates good consistency.

In order to evaluate the repeatability of ADC, we used the Bland–Altman method, which compares the 95% confidence interval (limit of agreement [LOAs]) between the first and second sets of DWI sequences and the mean absolute difference of ADC values. The median of image quality evaluation was obtained from the 3 b-value DWI images and ADC map. The inter-observer agreement for image quality was analyzed by calculating weighted kappa coefficients (quadratic weighting), with kappa values of 0.01–0.25 representing slight agreement, 0.25–0.45 fair, 0.45–0.65 moderate, 0.65–0.85 substantial, and 0.85–1.00 almost perfect agreement. The Friedman test was used to compare the differences between the six methods, and the Dunn-Bonferroni post-hoc test adjusted for all significant pairwise comparisons. Statistical analysis was performed using SPSS (version 19.0, Chicago, IL) software. When the P-value was less than 0.05, the difference is considered significant.

## Supplementary Information

Supplementary Information.

## Data Availability

All data generated or analysed during this study are included in this published article and its supplementary information files.

## Abbreviations

DWI: Diffusion-weighted imaging
ADC: Apparent diffusion coefficient
BH-DWI: Breath-hold DWI
FB-DWI: Free-breathing DWI
NT-DWI: Navigator-triggered DWI
RT-DWI: Respiratory-triggered DWI
RS-DWI: Readout-segmented DWI
Z-DWI: Zoomit DWI
c-mCNR: Cortico-medullary contrast to noise ratio
LOA: Limit of agreement
KC: Kidney cortex
KM: Kidney medulla
