# Supplementary material for: Comparing the clinical utility of single-shot, readout-segmented and zoomit echo-planar imaging in diffusion-weighted imaging of the kidney at 3 T
Source: Sci Rep. 2022 Jul 20;12:12389. doi: 10.1038/s41598-022-16670-w (PMC9300617; doi:10.1038/s41598-022-16670-w)
Supplement: Supplementary file 1 — Supplementary Information. [file 41598_2022_16670_MOESM1_ESM.pdf]

Comparing the clinical utility of single-shot, readout-segmented and zoomit echo-planar imaging in diffusion-weighted imaging of the kidney at 3 tesla

Wenguang Liu[1,2#], Hui Liu[1#], Simin xie[1], Ismail Bilal Masokano[3], Yu Bai[1], Xiao Wang[1], Linhui Zhong[1], Yi Wu[1], Jilin Nie[1], Gaofeng Zhou[1], Yigang Pei[1,2*], Wenzheng Li[1,2*]

# These authors contributed equally to this work

* These authors contributed equally to this work

1. Department of Radiology, Xiangya Hospital, Central South University, Changsha, Hunan, P.R. China, 410008

2. National Clinical Research Center for Geriatric Disorders, Xiangya Hospital, Central South University, Changsha, Hunan, P.R. China, 410008

3. Department of Radiology, The Third Xiangya Hospital, Central South University, Changsha, Hunan, P.R. China, 410013

* Corresponding author: Dr. Yigang Pei, street address: No.87 Xiangya Rd., Kai Fu District, Changsha, Hunan, P.R. China, 410008, telephone number: +86-15388927005, e-mail: xypyg0731@csu.edu.cn and Dr. Wenzheng Li, street address: No.87 Xiangya Rd., Kai Fu District, Changsha, Hunan, P.R. China, 410008, telephone number: +86-13574853656, e-mail: wenzheng727@163.com

Supplementary Table 1. The raw data of repeatability of ADC measurements

Volunteer 1		BH-DWI		FB-DWI		NT-DWI		RT-DWI		RS-DWI		Z-DWI	
		DWI	ADC	DWI	ADC	DWI	ADC	DWI	ADC	DWI	ADC	DWI	ADC
UP1	R1	46.89	1638.58	53.22	1621.39	86.33	1800	86.5	1682.13	137	2116.75	145	1093
	R2	33.67	1377.78	47.56	1437.56	86.44	1570.5	85.33	1512.44	140.33	1497.5	145.83	934.6
MP2	R3	51	1683.67	55.38	1831.22	83.11	1719	94.25	1737.89	142	1919	156.6	1262.2
	R4	38.67	1637.78	51.83	1509.22	89.38	1765.38	92.44	1664	163.5	1677.67	146.75	1178.5
LP3	R5	62.92	2048.75	75.89	1799.75	127.89	1822.3	124.75	1975.67	175.33	1883	177.75	1412.8
	R6	41.5	1716.88	56.75	1657.5	112.75	1721.43	109.9	1759.3	186	1619	159.5	1340.8
UP1	L1	31.75	2226.67	45.78	1623.56	73.44	2202.13	77.09	1745.18	122.5	2045	145.5	1027.8
	L2	40.2	1995	51.33	1680.4	81.13	1637.56	75.56	1747.33	134	1621	138.6	1001.8
MP2	L3	45.44	2080.3	72.8	1690.3	98.88	1887.8	99.63	1936	178	2028.67	188.5	1439.4
	L4	34.14	1797.22	62	1541.17	95.78	1547.11	102.88	1853.22	131	1570	173.8	1273.14
LP3	L5	45.78	2817.44	67.7	1498.11	100.58	1702.6	91.5	2104	171.5	1841.5	197.4	1359
	L6	34.13	1783	55	1467.56	95.89	1529.6	95.11	1515.33	157	1575.67	173	1343.6

Volunteer 2		BH-DWI		FB-DWI		NT-DWI		RT-DWI		RS-DWI		Z-DWI	
		DWI	ADC	DWI	ADC	DWI	ADC	DWI	ADC	DWI	ADC	DWI	ADC
UP1	R1	41.9	1913.13	38.18	1804.86	99.63	1984.78	99.5	1946.11	132.75	2146.67	208.33	1494.83
	R2	27.3	1638.58	32	1580.71	82.88	1775.8	92.7	1736.5	102.5	2027.33	191.83	1481.83
MP2	R3	50.56	1994.15	59.1	1939.75	115.14	1918.92	114.5	1956.79	153.67	2172.5	248.17	1705.5
	R4	39.38	1666.5	51.11	1883.86	107.25	1819.6	97.8	1832.08	129.67	2059.33	218.57	1600.5
LP3	R5	56.63	1903.4	47.7	2030.43	106	2030.2	100.25	2053.33	148.5	2360.67	201.8	1649
	R6	35.7	1900	39.5	1667	102.33	1924.25	77.11	1902.7	144.75	2199.5	198.75	1515
UP1	L1	37.44	1958.58	48.8	1903.25	77.89	1960.78	80.71	1954.2	103.5	2587	207.75	1542.33
	L2	27.3	1789.71	37.88	1866.9	65.75	1781.92	73.25	1681	73.67	2197.33	199.17	1538.43
MP2	L3	53	2024.1	71.08	2009.89	115.43	2002.56	125.86	1911	108	2551.25	272	1813.88
	L4	34.3	1660.71	47.7	1942.43	108.89	1909.56	100.6	1830.5	87.5	2351.5	237.25	1621.83
LP3	L5	71.56	1919.29	46.42	2374.5	99.33	2012.22	122.43	1960.18	99	3135.33	229.33	1841
	L6	39.9	1847.36	33.75	2037.7	96	1847.1	108.4	1770.58	75.75	2803	185.5	1642

Volunteer 3		BH-DWI		FB-DWI		NT-DWI		RT-DWI		RS-DWI		Z-DWI	
		DWI	ADC	DWI	ADC	DWI	ADC	DWI	ADC	DWI	ADC	DWI	ADC
UP1	R1	44.33	1617.38	53.89	1647.29	51.67	1719.22	84.5	1701.75	150.33	2124.5	193.17	940.17
	R2	31.75	1438.25	52.44	1491.88	46.63	1525.25	79.43	1621.22	142	1455.5	169.6	853.5
MP2	R3	63.8	1870.89	77	1521.3	84.22	1684.11	134.75	1828.22	204	1752	234.8	1376.75
	R4	48.33	1667.11	68.13	1454.22	65.33	1582.89	95.22	1680.67	169	1565.67	218.17	1224.75
LP3	R5	79.88	2738.78	94.33	1776.75	92.38	1819	1596	2732.75	219	1853	245	1367.17
	R6	44.13	1433.3	78.92	1532.56	74.56	1746.71	133.11	1730	195	1476.5	199.5	1157.25
UP1	L1	38.22	1866.56	50.7	1844.38	57.44	1685.92	95.33	1850.17	114	2519	192.5	1188.25
	L2	30.78	1557.75	45.58	1524.78	46	1410	78.56	1658.22	75	1672	171.4	659.5
MP2	L3	53.82	1838.4	72.83	1731.55	68.73	1817.56	122.33	1877	139	2255	248.71	1532.67
	L4	34.8	1566.67	63.5	1555.63	59.33	1507	87.78	1688.67	70	1926	203.33	1101.5
LP3	L5	60.5	1951.5	81.44	2333.14	86.78	1711.7	120	1783	135	2154.5	254.25	1383.8
	L6	34.78	1527.36	45.78	1564.2	67.25	1360.91	102.27	1593.7	132.67	1792	190.25	1072

Volunteer 4		BH-DWI		FB-DWI		NT-DWI		RT-DWI		RS-DWI		Z-DWI	
		DWI	ADC	DWI	ADC	DWI	ADC	DWI	ADC	DWI	ADC	DWI	ADC
UP1	R1	57.6	1877.58	55.38	1951	88.38	1602.5	86.11	1760.82	150	2039	165.75	1544.6
	R2	49.78	1746.33	48.22	1631	84.75	1616.13	88.11	1671.25	126.5	1652	134.25	1273.29
MP2	R3	61.1	1831.38	69	1841.3	100.5	1684.89	95.5	1766.33	165	1738	177.75	1531.83
	R4	53.78	1815.78	64.11	1648.67	96.7	1559.33	90.91	1748.56	151.5	1568.75	143	1419
LP3	R5	104.5	2244.83	87.56	1910.25	120.13	1986.56	119.88	1950.5	201	2247	193.2	1684.2
	R6	54.56	1789.75	56.56	1694.75	112.75	1722.44	102.67	1810.56	124	1694	154.4	1385.75
UP1	L1	49.56	2035.67	43.89	1886.67	71.11	1658.9	67.5	1855	114.33	1958	172.57	1313.83
	L2	38.56	1352.2	41.22	1643.4	62.25	1538.67	64.17	1574.91	109.33	1687	148.25	998.5
MP2	L3	61.22	1910.3	70.67	2032.3	109.67	1852.58	76.25	1975	179	2160	232	1694.17
	L4	40.78	1360.38	48.22	1572.13	78.89	1523.6	75.2	1784.33	141	1356.33	162.75	1321.2
LP3	L5	92.89	1876	75.78	1746.36	113.44	2269.88	101.63	2283.86	167	1991	220.25	1629.6
	L6	41.13	1490.5	54.89	1633.43	74.25	1647.78	85.89	1753.88	123.5	1604.5	168.67	1451

Volunteer 5		BH-DWI		FB-DWI		NT-DWI		RT-DWI		RS-DWI		Z-DWI	
		DWI	ADC	DWI	ADC	DWI	ADC	DWI	ADC	DWI	ADC	DWI	ADC
UP1	R1	36.43	2198.44	42.78	2226.89	88.13	2027	81.11	1928.17	134.5	2416	143.5	1556.25
	R2	24.63	1716.25	28.33	1986.25	71	1922.11	75.29	1699.29	86.5	163	118	1563.25
MP2	R3	44.25	2101.89	50.2	1789.25	98.89	2005.44	100.5	1856.11	142.5	2669	149.5	1672.75
	R4	35	1748.11	45.8	1773.75	74.33	186.56	88.25	1675.33	114	1995	146	1274.67
LP3	R5	40.9	2839.44	38.88	2057.75	103.75	2432.25	94	2241.33	149	1849	150	1805.5
	R6	22	1685.3	67.56	1696	66.67	1755.29	81.75	1768.5	88	2350.5	111.17	1153
UP1	L1	17.75	2561.11	34.56	2295.8	58.33	3138.3	61.22	2083.67	64.5	2606	114.8	1615.4
	L2	10.17	1618.44	23.1	1783.64	47.78	1842.9	53	1877.4	46	2116	99	1408.86
MP2	L3	27.33	2377.67	40.44	2145.1	74.7	2107.33	73.22	2010.89	108	2578	159.14	1904.2
	L4	15.22	1845.44	27.67	1736.63	61.39	1800.2	62.88	1471.38	70	2055.5	122	1382.78
LP3	L5	38.38	2680.75	44	1915.33	72	2228.22	76.88	2274.7	111	2310.5	145.17	1647.17
	L6	14.5	1795	19.3	1626.57	46.7	1863.4	56	1678.75	88.67	1878	118	1340.2

Volunteer 6		BH-DWI		FB-DWI		NT-DWI		RT-DWI		RS-DWI		Z-DWI	
		DWI	ADC	DWI	ADC	DWI	ADC	DWI	ADC	DWI	ADC	DWI	ADC
UP1	R1	33.13	2025.63	45.44	1964.22	69.75	2002.88	62.44	1769.2	98.5	2518	117	1588.75
	R2	23.44	1383.6	41.75	1596.63	61.5	1642.75	54.44	1607	106.5	1624.33	114	1264.6
MP2	R3	41.5	2068.67	56.89	1641.63	81.13	1798.57	87.78	1727.55	136.33	1965.67	138.5	1443.75
	R4	42.6	197.04	50.11	1270.29	50.88	1447.29	73.56	1466.44	107	1303.33	119	1151.67
LP3	R5	54.58	21.84.1	67.33	1809	106.88	1993	116.33	1778	157.5	2074.67	134.25	1577.2
	R6	33.55	1586.18	59.78	1581.43	77.86	1631.43	79.67	1563.33	81.5	1799	114.75	1032.25
UP1	L1	24.11	1845	36.7	2076.4	55.25	1856.22	54.38	1824.09	48	2372.5	131.5	1513
	L2	8.13	1358.63	19.89	1509.67	46.89	1600.5	48.89	1770.5	37	1591	103	1124.3
MP2	L3	47.22	1808.88	67.45	1815.44	99.14	1844.13	104.78	1725.3	118	2311	193.25	1779.4
	L4	23.88	1388.8	53.5	1436.86	51.43	1665.75	62.56	1608.77	82.5	1825	141.8	1291.4
LP3	L5	46.8	1828.6	58.17	2018.13	89	1996.67	107.89	1921.67	132.33	2088	170.75	1817
	L6	19.5	1701.36	32.33	1863.56	61.29	1731.29	73.5	1712.89	57.25	1755	124.2	1258

Volunteer 7		BH-DWI		FB-DWI		NT-DWI		RT-DWI		RS-DWI		Z-DWI	
		DWI	ADC	DWI	ADC	DWI	ADC	DWI	ADC	DWI	ADC	DWI	ADC
UP1	R1	39.57	1959.14	49.63	1956.33	73	1982.25	71.25	2102.25	123	2153	150.6	1571.75
	R2	17.33	1535.89	35.3	1640.89	42.71	1801.78	58.5	1734.63	87.5	1788	119.8	1158.5
MP2	R3	43.89	1882.29	55.5	1835.25	82.89	1906.57	82.63	2024.67	139.33	1981	176.33	1650.25
	R4	21.86	1548.17	43.9	1672.78	61	1735.25	59.14	1782.89	125	1924	132	1237.43
LP3	R5	52	2280.44	69	2019.25	94.29	2070	111.5	2013.56	157	1965.5	175.8	1727
	R6	32.89	1781.33	34.89	1823.89	83.5	1958.78	74.88	1796.67	137	1696.5	136.17	124.25
UP1	L1	27.33	1958.78	41	1845.44	63.78	1887.78	59.63	2011.5	97	2132	140	1512.75
	L2	16.9	1393.1	27.38	1746.89	48.25	1700.6	47.45	1823.22	72.5	1586	114.4	1065.8
MP2	L3	44.75	1927.89	58.78	1882.33	93.25	1957.63	90.11	1966.86	137.67	2099.67	175.6	1701.67
	L4	13.71	1697.75	27.11	1760.89	54.63	1730.44	65.08	1818	79	1696	114	1331.4
LP3	L5	61.43	1929.63	64.92	1730	112	2044.63	116	1965.56	180.67	1937.25	213.5	1736.25
	L6	28.1	1784.67	41.33	1560.22	60.36	1709.75	73.67	1895.71	126.75	1768	131.25	1383.2

Volunteer 8		BH-DWI		FB-DWI		NT-DWI		RT-DWI		RS-DWI		Z-DWI	
		DWI	ADC	DWI	ADC	DWI	ADC	DWI	ADC	DWI	ADC	DWI	ADC
UP1	R1	34.63	2066.5	48.2	1944.44	72.88	1798.22	75.25	1923.67	99.5	2202.25	133.25	1336
	R2	25.38	1580.22	34	1657	62.56	1627.45	65.88	1710.22	92	1008.25	125	1154.5
MP2	R3	49.78	2110.4	58.58	2014.5	81.6	1934.89	90.14	1822.89	124.5	1996.33	140.25	1431.5
	R4	30.78	1247.88	52.63	1718.89	73.38	1540.17	84.86	1632.89	103	1646	132	1063.83
LP3	R5	54.3	1910	66	2097.25	111	1920.25	110.43	1881.33	144.5	2225.5	154.75	1602.5
	R6	37.9	1512.38	41.9	1675.6	88.5	1713.9	103.13	1772.6	115	1928	126	1077.5
UP1	L1	36	1785.75	35.63	1721.25	71.2	1766.83	72.5	1926.6	118	2002	133.14	1409.17
	L2	21.56	1284.89	27.75	1512.8	47	1517.38	51.63	1498.89	72	16007.5	125.14	1101.83
MP2	L3	42.13	2118	56.22	2031.33	85.22	1894.89	128.13	1629.63	162	2007	198.2	1746.33
	L4	16	1222.45	36	1471.22	51.5	1516.33	59	1474.13	104	1712	139	1333.5
LP3	L5	54.56	1821.38	57.75	2182.22	86.5	1715.89	82.89	1909.9	143.5	1948	160	1697
	L6	24.5	1343.83	33.78	1536.33	65.11	1573.6	81.25	1570.22	102.25	1645.33	133.6	1178.71

Volunteer 9		BH-DWI		FB-DWI		NT-DWI		RT-DWI		RS-DWI		Z-DWI	
		DWI	ADC	DWI	ADC	DWI	ADC	DWI	ADC	DWI	ADC	DWI	ADC
UP1	R1	30.25	1968	50.09	1989.11	61.33	2095.67	50.67	2414.17	110.5	2130.33	171.25	1711.5
	R2	17.6	1608.63	37.75	1622.89	43.44	1860.75	33.5	1990.4	82.5	1778.5	133.5	1492.17
MP2	R3	39.56	2005.33	53.3	1966.4	75.08	2031.11	70.1	1999.11	110.5	2199.33	176.8	1871.25
	R4	28	1585.44	45.89	1690.89	73.33	1631.75	62.63	1701.89	83.75	1853	138.71	1511.6
LP3	R5	71.25	2234.6	72.22	2045	96.9	2094	98.89	2014.3	129.5	2287	203.5	1902.6
	R6	29.78	1669.78	51.67	1669.78	56.56	1629.75	65.13	1755.78	83	1955	160.2	1684.83
UP1	L1	28.11	1787.78	43	1835.7	59.78	1863.4	62.33	1869.88	112	2017.67	170.25	1771
	L2	25	1486	31	1565.5	52.08	1604.56	38.33	1506	87	1607.5	163	1515.71
MP2	L3	37.7	1836.64	58.67	1921.63	92.25	1985.5	87.11	1852.11	141.5	1839.5	230.17	1921.86
	L4	31	142.44	42.44	1473.22	42.33	1564.89	40.58	1542.5	98.5	1510	176	1581.75
LP3	L5	37.44	2086.78	50.25	1768.75	80.75	1911.71	75.11	1757.56	150	1829.5	225.25	1760.5
	L6	28.78	1573.89	47.22	1643.1	64.88	1571.86	59.25	1516.88	123	1541	187.5	1485.6

Volunteer 10		BH-DWI		FB-DWI		NT-DWI		RT-DWI		RS-DWI		Z-DWI	
		DWI	ADC	DWI	ADC	DWI	ADC	DWI	ADC	DWI	ADC	DWI	ADC
UP1	R1	57	2020.56	73.78	2036.56	115.44	1923.5	120.13	1702	166	2215	173	1098.5
	R2	38.33	1616.23	61.1	1665.88	100.2	1679.78	112.44	1618.1	136	1967	166.5	1021.17
MP2	R3	69.88	1680.5	84.67	1801.11	129.56	1815.67	133.8	1698.8	222.5	1845.33	207	1425.5
	R4	46.44	1321	70.2	1664	117.29	1593.38	125.6	1500.5	190.67	1531	188.75	1176.5
LP3	R5	48.25	1968.13	107.08	1741.38	135.38	1969	163.29	1607.1	164.5	1884	176.75	1176.83
	R6	30.78	1371.75	91.78	1524.5	121.18	1606.86	129.75	1557.08	122	1744	170.75	109.25
UP1	L1	4089	1781.5	48.33	1798.78	70.13	1899.25	68.44	1742.14	130.5	1803.67	167	1239.86
	L2	32.5	1507.1	30	1537	58	1562.11	65.89	1553.67	120.5	1264.33	152.67	1120.75
MP2	L3	60.63	1786.78	92.5	1774.56	133.63	1759.2	130.2	1652.63	242.5	1710.5	224	1482.86
	L4	45	1202.86	56.1	1511.75	89	1621.38	94.25	1470.67	126.5	1256	201.67	1271.43
LP3	L5	67.1	1791.78	89.1	1835.3	146.63	1828.38	149.29	1711.45	200.5	1759	205.8	1405
	L6	39.88	1500.67	63	1479.11	82.8	1489.5	90.75	1545	152	1353	170.75	1132

Volunteer 11		BH-DWI		FB-DWI		NT-DWI		RT-DWI		RS-DWI		Z-DWI	
		DWI	ADC	DWI	ADC	DWI	ADC	DWI	ADC	DWI	ADC	DWI	ADC
UP1	R1	57.67	1637.22	71.22	1643.8	115	1772.22	119.5	1721.11	186.5	2163	195.33	1166.75
	R2	47.33	1210.22	57	1324.75	87.67	1732.86	102.86	1477.25	154.75	1517	155	1154.25
MP2	R3	59.75	1877.89	89.44	1780.5	123	2028	150.25	1697.89	225.33	1986	256	1480.5
	R4	58.29	1464.6	76.33	1588.89	86.43	1643.88	144.22	1496.63	195	1659	217.5	1293.75
LP3	R5	66.14	2258.6	61.11	1692.89	132.13	1857.75	136.77	1964.67	190.5	2055	210.67	1431.2
	R6	48.38	1549.21	74.44	1454.78	111.89	1509.22	132.22	1607.55	162	1521	205	1294.33
UP1	L1	45.1	1889.75	54.56	1720.22	90.75	1885.22	94.22	1869	159.5	1847	21.33	1368.8
	L2	41	1521.2	47.29	1515.11	65.5	1664.75	83.38	1613.22	147	1420	193.8	1127.25
MP2	L3	70.33	1901.83	96.63	1853.75	131.2	1934.11	135.5	1794.23	229.5	1739	251.8	1649.5
	L4	59.2	1657.75	73.11	1543.22	99.33	1509.33	96.78	1628.56	171	1477	227.5	1324.83
LP3	L5	96.11	1564	76.67	1744.14	135.5	1816.13	153.33	1984	213	2030	259.5	1503.25
	L6	48	1460.25	61.56	1358	98.13	1537.25	111	1609.71	187	1666.76	191.8	1364.25

Volunteer 12		BH-DWI		FB-DWI		NT-DWI		RT-DWI		RS-DWI		Z-DWI	
		DWI	ADC	DWI	ADC	DWI	ADC	DWI	ADC	DWI	ADC	DWI	ADC
UP1	R1	46.17	1801.17	52.88	1985.8	106.71	1819.29	87.43	179838	113	2266	158.25	1212
	R2	33.33	1617.43	43.75	1548.88	88.92	1562.71	80.38	1603.29	99	1825	144.25	1189.6
MP2	R3	46.22	1757.45	61.2	1890.88	108.5	1878.68	98	1853.71	125.5	2349	178.25	15004.5
	R4	37	1473.67	59.3	1787.78	88.33	1618.88	84.63	1504.33	95	1969.5	154.8	127.4
LP3	R5	54.25	1926.6	67.2	2037.78	135.5	1845.8	121	1896.25	161	2319.33	200	1555.75
	R6	38.6	1534.67	57.71	1700.63	112.56	1770.56	109.63	1676.89	123	1770.5	168.2	1378
UP1	L1	32	1870	46.89	1806.89	73.78	1861.11	58.6	1793.57	86.67	2021	142.75	1133.4
	L2	23.67	1616.83	40.44	1553.5	68.63	1636	56.88	1481.5	78.5	1662	132.5	952
MP2	L3	54	1790.6	60.83	2027.33	93.92	1886.92	98.67	1914.17	138.5	2133.5	205	1607.57
	L4	32.18	1666.38	49.38	1693	83.38	1721	92.3	1514.78	131.5	1774	177.75	1327
LP3	L5	49.29	2076.75	72.6	1917.22	119.33	1821.88	114.89	1845.7	160	1982	200.75	1493.5
	L6	40.71	1532	54.78	1652.38	110.13	1763.55	86.13	1581.78	123	1813	177.33	1251.25

Volunteer 13		BH-DWI		FB-DWI		NT-DWI		RT-DWI		RS-DWI		Z-DWI	
		DWI	ADC	DWI	ADC	DWI	ADC	DWI	ADC	DWI	ADC	DWI	ADC
UP1	R1	36	1782.14	40.22	2065.25	68.17	2047	78.89	2246.33	90	2650	131.5	1386
	R2	31.43	1679.33	33.86	1767.5	64.22	1931.5	73	1946	65	1967	127.5	1385.4
MP2	R3	37.71	1911.78	50.67	1976.56	85.75	1964.86	87.83	2055.44	112.5	2555.5	155	1675.75
	R4	28.14	1747	38.75	1831.57	65.83	1839.57	76.38	1740.5	99.5	2095.5	139.75	1494.75
LP3	R5	43	1538.43	59.13	2001.22	97.22	2007.33	97.89	2098.27	115.67	2393	178.25	1752
	R6	27.71	1503	38	1775.2	83.6	1937.5	89	2016.11	104	2113	149.33	1688.75
UP1	L1	18.27	1695.75	21.8	1874.71	26.75	2009.63	45.64	2158.78	64.67	2189	111.25	1084.83
	L2	11	1447.57	20.13	1608.38	29	1847.38	37.09	1806.7	63	1925.5	107.5	1137.87
MP2	L3	27.88	1929.63	37.5	1906	55.17	2252.83	77.5	2115	99.5	2045.67	138.8	1622.14
	L4	21.11	1812.78	29.75	1852.38	35.5	2073.5	65.25	1824.67	85.5	1711.5	121.25	1598
LP3	L5	37.39	1684.29	32.43	1802.25	87.33	2192	86.13	2008.5	127.25	1940	149.86	1718.17
	L6	28.27	1600.57	30.15	1575	54	1878.88	65.4	1805.11	115	1851	137.33	1555.83

Volunteer 14		BH-DWI		FB-DWI		NT-DWI		RT-DWI		RS-DWI		Z-DWI	
		DWI	ADC	DWI	ADC	DWI	ADC	DWI	ADC	DWI	ADC	DWI	ADC
UP1	R1	29.45	2054.29	39.13	1461.11	63.56	2088.29	50.78	1472.38	81	2557	137.67	1335.56
	R2	21	1307.78	29.22	1439.6	54.22	1578.78	52.13	1549.56	72.5	2231	113.8	1027.83
MP2	R3	38.25	2146	40.22	1396.38	75.13	2129.14	56.88	1867.67	99	2665.5	132.5	1349
	R4	27	1408.38	39.13	1471	55.86	1647.38	42.6	1767.29	71.67	1797.5	120.2	1162.67
LP3	R5	51.8	1817.38	61.38	1942.7	95.88	2084.25	77.3	2553.67	141	2581.5	154.4	1520.5
	R6	25.56	1554.63	37.8	1281	64.5	1799.5	69.75	1788.5	86	1776	119.67	1409.83
UP1	L1	22.5	2030	30.56	1657.3	38.08	1961.5	34.5	1654.89	66.33	1926.5	121	1274.75
	L2	13.11	1357.7	23.67	1434.29	30.13	1861.44	33.33	1545.22	62.5	1542.33	108.5	1109.67
MP2	L3	43.78	2058.58	56.22	1824	89.5	2016.88	79	1941.4	139.5	2057	147.25	1601.83
	L4	24.63	1573.09	40.11	1665.7	53.9	1696.5	35.63	1413.5	88.5	1694	116.75	1193.75
LP3	L5	44.22	2246.33	66.7	1792.89	90	1987.75	76.88	2414.22	164.5	2006.5	180.86	1711.25
	L6	17.63	1487	43.56	1286.75	46.78	1792.8	57.25	1243.9	106.25	1611.5	104.83	1006.33

Volunteer 15		BH-DWI		FB-DWI		NT-DWI		RT-DWI		RS-DWI		Z-DWI	
		DWI	ADC	DWI	ADC	DWI	ADC	DWI	ADC	DWI	ADC	DWI	ADC
UP1	R1	49.3	1523.63	69.63	1938.89	114	1645.58	94.57	1917.25	182	1827.67	169.6	1374.5
	R2	50.13	1600.33	63.4	1559	10.56	1751.56	92.38	1875.75	168.5	1612	167	1361.75
MP2	R3	57.43	1703.67	72.89	1537.11	105	1821.25	112.75	1690.63	173	1644	193	1441
	R4	45.58	1639.38	60	1472	96.88	1662.44	109.33	1687	158.33	1511.5	178.83	1457.67
LP3	R5	80.25	2334.83	82.75	3062.13	134.4	2332.38	127.22	2655.89	180.33	1950.5	217.8	1569
	R6	42.8	1710.22	58.75	1699.5	91.67	1687.43	93	1656.33	172	1747.67	153.2	1375.75
UP1	L1	66.13	2019.78	48.25	1743.86	84.67	2122.44	88.5	1922.67	154	1915.33	168.8	1346.43
	L2	36.67	1648.67	42.64	1770.78	64.25	1838	71.13	1870.33	128	1672.67	152	1314.2
MP2	L3	51.67	1880.75	63.11	1874.33	94.42	1938.63	100.88	1841.67	164.5	1878.25	221	1634.83
	L4	38.67	1680	49	1618.22	82	1851	78.75	1542.22	131	1720	190.5	1564.17
LP3	L5	58.5	2093	63	1992.17	104	2011.8	101.7	2515.44	180.33	2148.75	243.4	1853.4
	L6	31.33	1403	41.91	1380.8	82.38	1805.89	90.75	1629.88	106.5	1426	159	1317.67

Volunteer 16		BH-DWI		FB-DWI		NT-DWI		RT-DWI		RS-DWI		Z-DWI	
		DWI	ADC	DWI	ADC	DWI	ADC	DWI	ADC	DWI	ADC	DWI	ADC
UP1	R1	40.11	2114.75	56.57	2153.29	82.22	1967.88	70.33	2093.67	144.5	2355	157.75	1389.25
	R2	31.22	1247.78	34	1666.6	76.14	1738.44	71.89	1845.18	100	1769.67	132.17	1358.6
MP2	R3	44.56	201.44	57.45	1897.1	81.38	1963.11	79.22	2039.25	136	2041	175.83	1600.83
	R4	42.78	1736.33	42.38	1646	69.5	1711.13	66.67	1971.14	86	1746	163	1307
LP3	R5	54.33	2329.71	69.83	1955.9	100.4	2035.89	94.5	2048.56	152.5	2137	179.2	1593.8
	R6	33.4	1601.9	40.25	1931	59.25	1967.63	89	1865.44	89	1919.5	140.4	1448.8
UP1	L1	30.67	2248.2	42.33	2077.33	64.5	1982.56	58.22	1893.88	95	2460.5	140.5	1338.2
	L2	20.5	1882.13	32.38	1592.88	59.89	1823.78	51.88	1778.75	81	1857	137.6	1116.75
MP2	L3	51.67	1896.44	66.45	1957.3	87.13	2017	105.25	1967.56	145	2086.5	172.5	1553.83
	L4	26.88	1399.14	46.11	1612.67	63.3	1629.9	75.63	1765.89	117	1717	156.17	1169.5
LP3	L5	48.33	2243.43	76	1866.88	97.5	2063.78	115.67	1857.3	169	2233	183	1736.6
	L6	34.67	1664	49.38	1792.78	57.67	1809.88	112.43	1751.13	116.5	1627	155	1435.17

Volunteer 17		BH-DWI		FB-DWI		NT-DWI		RT-DWI		RS-DWI		Z-DWI	
		DWI	ADC	DWI	ADC	DWI	ADC	DWI	ADC	DWI	ADC	DWI	ADC
UP1	R1	59	1726.5	71.17	1602.17	116.5	1683.3	122.63	1713.38	191.5	1868.5	212.33	1333.33
	R2	43.38	1424.45	68.33	1553.57	107.5	1530.75	119.38	1501.6	185	1385.5	187	1253.5
MP2	R3	58	1664.67	71.67	1719.63	113.14	1787.22	115.5	1768.7	186	1780.5	229.5	1410.75
	R4	50	1339.14	67	1632.86	104.4	1540.63	112.71	1495.75	173.5	1113.5	177.5	1170.25
LP3	R5	71.7	1934.4	92	1843.86	136.11	1749.5	164.86	1609	211	1895	239.5	1510.5
	R6	46.56	1457.8	58.5	1416.5	93.63	1517	96.25	1508.44	180	1522.5	229.5	1239.67
UP1	L1	38.14	1783.83	48.57	1789.57	68	1858.64	82	1607.11	141	1885.5	190.5	1273
	L2	35.2	1489.5	44.33	1591.43	63.13	1683.36	78.13	1536.44	133	1440.5	179.75	1188.67
MP2	L3	65.5	1797.86	84.2	1825.75	114	2010	125.57	1618.33	235	1800	277.5	1602.5
	L4	31.78	1476.63	72	1057.29	103	1608.78	120.88	1435.75	178.5	1330	235.25	1312.33
LP3	L5	87.71	1973.43	88.13	1859.88	132	1928.08	132.6	1717.56	201.5	1765	246.2	1486.33
	L6	40.29	1133.13	70.5	1485.33	96.14	1608.33	123	1455.25	189	1448	210.33	1233.67

Volunteer 18		BH-DWI		FB-DWI		NT-DWI		RT-DWI		RS-DWI		Z-DWI	
		DWI	ADC	DWI	ADC	DWI	ADC	DWI	ADC	DWI	ADC	DWI	ADC
UP1	R1	46.4	1706.25	54.56	1787	86.6	1687.75	94.33	1971.78	154.1	2141	149.5	1201
	R2	24.11	1569.38	40.13	1497.13	63.63	1746.22	84.43	1745.22	85	1878.5	128.25	1122
MP2	R3	50.89	1888.78	59.63	1794	92	1855.8	96.33	2061.6	125	2307	174.2	1536.5
	R4	37	1457.3	51.55	1667.25	72.13	1597.75	86.4	1790.18	91.5	1985	141.25	1260
LP3	R5	55.25	1859.58	71.44	2074.43	104.25	1856.71	106.25	2071	145	2398	169	1439.25
	R6	39.9	1417.91	47.11	1707.86	69.75	1691	78	1756.56	121	1982	153.25	1307.67
UP1	L1	31.42	2432.63	49.17	1794.86	77.11	1728.13	85.88	2226.4	136	2174.5	149	1388.33
	L2	18.25	1620.78	36.17	1499.29	64.33	1594.8	64.43	1739.83	91	1842	126.5	1093
MP2	L3	50.56	2226	71.22	2091.89	103.56	1952.56	110.38	2114.11	175.67	2256	200.67	1726.6
	L4	22.67	1921.2	46.1	1542.43	75.67	1493.5	80.49	1844.67	132	1883	166.5	1144.25
LP3	L5	41.44	2163.69	78.75	2419.33	124	1993.5	129.71	2017.25	192	2547	203.33	1594
	L6	14.27	1956.44	41.1	1355.1	81	1591.7	84.33	1776.25	92	1630	151	1353.25

Volunteer 19		BH-DWI		FB-DWI		NT-DWI		RT-DWI		RS-DWI		Z-DWI	
		DWI	ADC	DWI	ADC	DWI	ADC	DWI	ADC	DWI	ADC	DWI	ADC
UP1	R1	36.14	2102.44	45.5	1819.67	62.71	2187.43	69	2086.86	109	2334.5	141.67	1518.25
	R2	18	1319.43	32	1779.43	50.86	1712.63	67.14	1800.25	84.5	1904	133.75	1104.5
MP2	R3	45.86	1920.11	55	1966.86	68.38	2170.86	80.38	2058.14	104.5	2275	165	1508.5
	R4	31.22	1566	40.14	1759.86	62.43	1839.33	70.75	1804.43	78.5	1853.75	144.9	1353
LP3	R5	57.63	2129.75	73.83	1757.33	106.38	2319.5	113.83	2042.13	175.5	2179.5	171.25	1488.6
	R6	44.29	1584.86	44.13	1662.5	96	1836.67	68.5	1783	144	1722	150.75	1432
UP1	L1	27.22	2108.89	21.22	1762.5	35.8	2122.88	27.33	2076.14	60	2163	139.75	1442.5
	L2	8.71	1462	14.5	1712.5	33	1867.86	26.71	1698	34	2051.67	129.25	1173.75
MP2	L3	48.14	2073.78	57.38	2023	79.75	2156.75	90	2042.56	151	2356	201.4	1735.25
	L4	22.88	1774.22	33.86	1655.86	57.88	1773.11	58.71	1521.17	87	1696.5	150.6	1335.2
LP3	L5	64.29	1911.67	55	1923.29	94.71	2187.14	101.5	1958.71	170	2215.2	171.83	1659.25
	L6	27.88	1623.14	36.29	1732.86	70	1821	78.67	1607.29	123.5	1602	150.4	1334.25

Volunteer 20		BH-DWI		FB-DWI		NT-DWI		RT-DWI		RS-DWI		Z-DWI	
		DWI	ADC	DWI	ADC	DWI	ADC	DWI	ADC	DWI	ADC	DWI	ADC
UP1	R1	38.1	1707.92	41.67	2046.78	65.63	1746.63	76.25	1726	74	2152.33	175.23	1236
	R2	31.33	1475.44	30.78	1789.88	59.91	1559.5	74.88	1680.56	56.67	2174	124.3	1146.5
MP2	R3	46.17	1652.25	52.22	1817.56	96.56	1802.63	95	1817	105.33	2018	168	1563.5
	R4	34.73	1394.1	41.78	1609.69	78	1563.6	76.5	1637.3	86.3	1874.25	114.2	1203.75
LP3	R5	55.4	1721.82	50.25	1834.67	97.23	1808.38	108.25	1734	111	2287	186.67	1453.5
	R6	31.13	1557	43.38	1748	94.2	1575.69	99.86	1542	97.5	2020.5	123	1216
UP1	L1	30.7	1587.75	3867	1790.22	51.2	1831.67	60	1781.33	93.5	1717.5	184.4	1546.3
	L2	20.67	1395.44	30.33	1550.8	43.33	1632.44	50.78	1583.92	82	1367.89	132.2	1233.1
MP2	L3	51.22	1572.92	69.78	1776.11	107.33	1785.55	105.57	1794.33	187.5	1692.67	153.63	1563.5
	L4	35.44	1552	50.33	1666.25	92.56	1581	92.83	1481.4	132.5	1230	133.5	1250
LP3	L5	55.4	1603.89	57.13	1836.25	105.7	1799.58	107.13	1778.23	188	1699.5	174	1459.8
	L6	35.63	1491	43.71	1751.63	88.38	1673.78	90.56	1651.5	133.5	1370.67	122.4	1300

Volunteer 21		BH-DWI		FB-DWI		NT-DWI		RT-DWI		RS-DWI		Z-DWI	
		DWI	ADC	DWI	ADC	DWI	ADC	DWI	ADC	DWI	ADC	DWI	ADC
UP1	R1	53.11	1810.43	29.1	1896.92	59.89	1869.56	89	1816.38	163	1792	168.86	972.6
	R2	33.27	1561.4	24.33	1581.5	55.6	1662.78	81.11	1630.7	131	1617	160.67	922
MP2	R3	54.4	1764	45	2009.14	74.38	1858.11	104.33	1906.5	175.33	1894.5	198	1259
	R4	46.42	1575.3	34.22	1781.3	62.44	1600.3	96.86	1792.13	148.67	1447.5	175	1142.9
LP3	R5	61.63	1817	38.78	1873.4	74.88	1841	114.14	1942.71	183	1883.67	183.25	1255.4
	R6	38.75	1624.56	33.88	1649.69	57.25	1702.6	88.57	1723.89	143	1586	170	114.17
UP1	L1	44.36	1764	28.63	1809.43	56.63	1803	94.14	1925.63	151.5	1861.67	168.33	1140.75
	L2	26.63	1667.89	27.22	1607.89	49.1	1706.78	71.43	1721.8	121	1600	153.83	973.33
MP2	L3	68.63	1917.1	50.73	1930.89	85.92	1886.5	138.67	1918.44	186	1805	210.6	1410.14
	L4	41.88	1452.67	34.92	1471	63.78	1616.11	102.75	1754.78	135	1420	164.4	1146.88
LP3	L5	65.56	1676.2	50.44	1802.89	86.33	2055.25	145.38	1844.57	151	1892.33	211	1505.83
	L6	33.67	1422.88	41.1	1469.58	66.1	1779.75	115.57	1825.67	213.5	1636.33	186.75	1266.67

Volunteer 22		BH-DWI		FB-DWI		NT-DWI		RT-DWI		RS-DWI		Z-DWI	
		DWI	ADC	DWI	ADC	DWI	ADC	DWI	ADC	DWI	ADC	DWI	ADC
UP1	R1	39.63	1656	67.11	2293.25	85.8	2226	118.88	1850.75	137.5	1626.5	216.75	1265
	R2	32.14	1755.78	65.22	1575.89	80.64	1876	109.89	1713.11	129.5	1340	191	1055.5
MP2	R3	42	2130.38	80.75	2067.13	104.13	2130.22	151.4	1714.11	143.33	1761.5	241.2	1508.6
	R4	32.13	1319.56	56.78	1594.5	91.4	1766.13	124.11	1565.25	124	1596	208.75	1209.25
LP3	R5	56.13	1424.22	83.82	2500.5	129.75	2025.67	159.56	1756.25	158	2028	215.25	1307.4
	R6	32.13	2332.08	58.38	2082.38	87.78	1953.1	125.78	1253.67	103	1517.5	197.75	1125.17
UP1	L1	44.63	1802.67	30.22	2550.5	36	2253	59.44	1853.56	153.5	1803.5	202	1202.1
	L2	31.5	1598.64	29.38	1365	39.7	1829	45.5	1486	105.5	1512	177.5	1000
MP2	L3	49.22	1875.38	83.38	2344.38	106.67	2143.89	136	1819.58	173	1880.75	250	1567.5
	L4	36.57	1569.8	48	1133	71.33	1782.11	108.11	1465.56	124	1348.5	190.25	1240.86
LP3	L5	32.88	1713.63	76.38	2146.56	98.9	2023.67	157	1820.56	198	1937	222.86	1650
	L6	63.4	1605.88	57.22	1708.78	69.44	1806.33	118.56	1598.38	147	1508.5	186.17	1280.86
